# Sub-minute Phosphoregulation of Cell Cycle Systems during *Plasmodium* Gamete Formation

**DOI:** 10.1016/j.celrep.2017.10.071

**Published:** 2017-11-14

**Authors:** Brandon M. Invergo, Mathieu Brochet, Lu Yu, Jyoti Choudhary, Pedro Beltrao, Oliver Billker

**Affiliations:** 1 European Molecular Biology Laboratory, European Bioinformatics Institute (EMBL-EBI), Hinxton, Cambridgeshire CB10 1SD, UK; 2 Malaria Programme, Wellcome Trust Sanger Institute, Hinxton, Cambridgeshire CB10 1SA, UK; 3 Proteomics Mass Spectrometry, Wellcome Trust Sanger Institute, Hinxton, Cambridgeshire CB10 1SA, UK; 4 Department of Microbiology & Molecular Medicine, CMU, University of Geneva, 1211 Geneva 4, Geneva, Switzerland; 5 The Institute of Cancer Research, Chester Betty Laboratory, London, Greater London SW7 3RP, UK

**Keywords:** gametogenesis, proteomics, signal transduction, ARK2, CRK5

## Abstract

The transmission of malaria parasites to mosquitoes relies on the rapid induction of sexual reproduction upon their ingestion into a blood meal. Haploid female and male gametocytes become activated and emerge from their host cells, and the males enter the cell cycle to produce eight microgametes. The synchronized nature of gametogenesis allowed us to investigate phosphorylation signaling during its first minute in *Plasmodium berghei* via a high-resolution time course of the phosphoproteome. This revealed an unexpectedly broad response, with proteins related to distinct cell cycle events undergoing simultaneous phosphoregulation. We implicate several protein kinases in the process, and we validate our analyses on the plant-like calcium-dependent protein kinase 4 (CDPK4) and a homolog of serine/arginine-rich protein kinases (SRPK1). Mutants in these kinases displayed distinct phosphoproteomic disruptions, consistent with differences in their phenotypes. The results reveal the central role of protein phosphorylation in the atypical cell cycle regulation of a divergent eukaryote.

## Highlights

• A sub-minute phosphoproteomic time course of *Plasmodium* gametogenesis is presented
• Hundreds of proteins are phosphoregulated within 20 s of gametocyte activation
• Replication- and mitosis-related proteins are phosphoregulated simultaneously
• Gene deletion reveals distinct roles for the protein kinases CDPK4 and SRPK1

## Graphical Abstract

**Figure:**
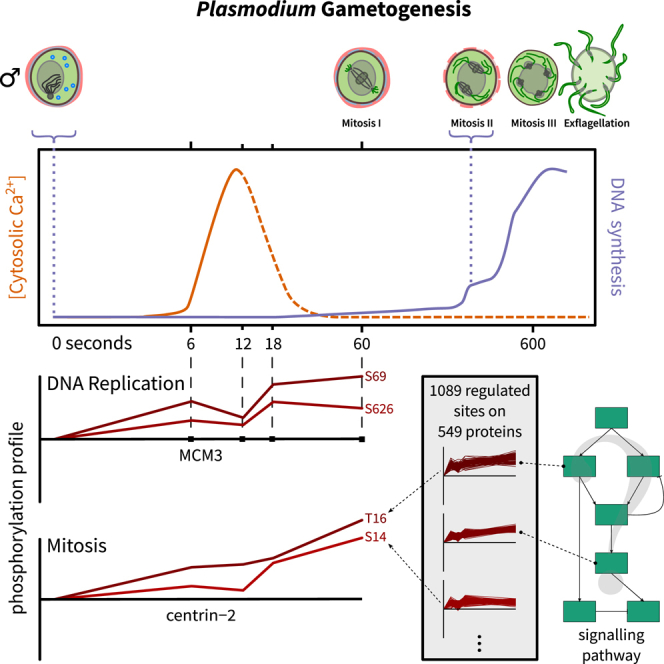

## Teaser

Invergo et al. measure a phosphoproteomic time course during a life cycle transition of a malarial parasite. They observed broad phosphoregulation on a sub-minute scale, including simultaneous regulation of replication- and mitosis-related proteins. Their analyses reveal conserved phosphorylation patterns, and they highlight functional roles of specific protein kinases during this process.

## Introduction

Malaria represents a major global health concern, causing an estimated 212 million cases resulting in approximately 429,000 deaths in 2015 (World Health Organization, 2016). It is caused by intracellular parasites of the genus *Plasmodium*, whose complex life cycles involve mosquito vectors of the genus *Anopheles* transmitting the parasites between vertebrate hosts. We lack fundamental knowledge about the molecular systems that regulate parasite development during transmission to the vector, which is initiated when developmentally arrested sexual precursor stages, the gametocytes, are ingested by a susceptible mosquito. Micro- and macrogametocytes respond to a small mosquito molecule, xanthurenic acid (XA), which must coincide with a drop in temperature to trigger the emergence of intraerythrocytic gametocytes and their differentiation into male microgametes and female macrogametes (Billker et al., 1997). Cytosolic Ca^2+^ levels rise steeply after a lag phase of 6–8 s and peak within the first 20 s of activation (Billker et al., 2004). Within 15 s, a microtubule-organizing center gives rise to eight kinetosomes in microgametocytes (Sinden et al., 1976). As soon as 60 s after activation, they have assembled the first mitotic spindle and four axonemes start to grow on the templates of kinetosomes at each spindle pole (Billker et al., 2002). Within 8–10 min, each microgametocyte replicates its genome three times, undergoes three rounds of endomitosis, and assembles eight axonemes in order to produce eight microgametes. The microgametes then extrude from the gametocyte in a flagellar manner, through a process called exflagellation. Fertilization of the macrogametes results in the development of motile ookinetes, which escape the mosquito midgut.

The signal transduction pathway controlling gametocyte activation is a validated target to block parasite transmission to the mosquito (Ojo et al., 2012). A more detailed understanding of the mechanisms that link extracellular triggers to the cell and developmental cycle is therefore of significant interest. Following activation, the cyclic guanosine monophosphate (cGMP)-dependent protein kinase PKG (McRobert et al., 2008) and the Ca^2+^-dependent protein kinase CDPK4 (Billker et al., 2004) become active, with PKG activity controlling the release of intracellular Ca^2+^ stores (Brochet and Billker, 2016). CDPK4 is essential for male gametogenesis. It controls DNA replication by regulating the assembly of the pre-replicative complex (Billker et al., 2004, Fang et al., 2017); it is further required early during microgametogenesis for the first mitotic spindle to form, and later for cytokinesis to occur and axoneme motility to become initiated (Fang et al., 2017). Other protein kinases and phosphatases required at various stages of gametogenesis include CDPK1 (Sebastian et al., 2012); a mitogen-activated protein kinase, MAPK2 (Tewari et al., 2005, Kern et al., 2014); and two protein phosphatases, PPM1 (Guttery et al., 2014) and the Ca^2+^-dependent calcineurin A (CnA) (Philip and Waters, 2015). The signaling relationships among these proteins remain unknown.

A comparison of phosphorylation events in a CDPK4-knockout mutant has identified a relatively small number of only 70 phosphosites that, within 18 s of gametocyte activation, differ significantly from wild-type in their phosphorylation state (Fang et al., 2017). A more comprehensive and unbiased network of co-regulated proteins can be obtained from observing the kinetics of phosphorylation (Kanshin et al., 2015). We have therefore constructed a high-resolution phosphorylation time course for the first minute of gametocyte activation. We exploit the advantages offered by *Plasmodium berghei*, a parasite specific to rodent hosts, which allows gametocytes to be readily purified in sufficient quantities for biochemical investigation, and whose purified gametocytes can be activated rapidly and effectively *in vitro*, in a highly synchronous manner. Our results suggest that, in addition to CDPK4, other lipid and protein kinases contribute to the early gametogenesis signaling response. We observed nearly instantaneous and simultaneous phosphoregulation of proteins underlying processes that are fundamental to gamete formation during the first seconds of cell activation, particularly the assembly of axonemes, the formation of the mitotic spindle, and DNA replication initiation and replication. We validated our analysis using the phosphoproteomes of both CDPK4 and SRPK1 mutants, and we further identified a new role for the latter in gametogenesis. We also present a resource of hundreds of regulated phosphosites of likely importance for controlling basic biological processes in malaria parasites.

## Results

### Robust Quantification of Phosphosites

**Figure 1:**
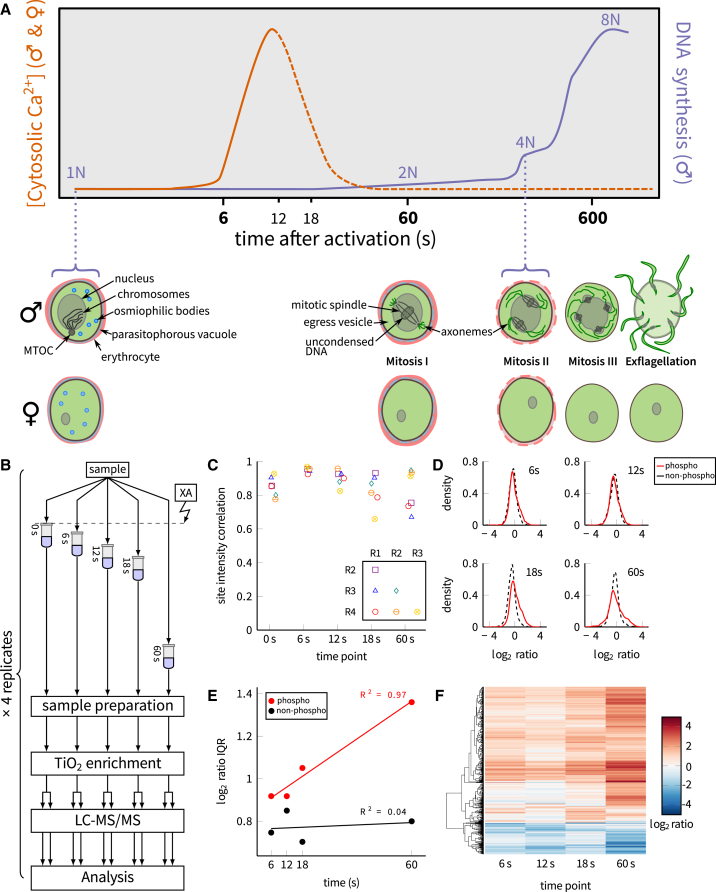
A Time Course of Phosphorylation Events during Gametocyte Activation (A) Schematic illustrating the timing of key events during *P. berghei* gametogenesis (MTOC, microtubule organizing center). (B) A schematic of the experimental design. (C) Correlation coefficients for phosphosite intensities among biological replicates for each time point (R1, replicate 1; R2, replicate 2; R3, replicate 3; and R4, replicate 4). (D) Kernel density distributions of *log*_2_ ratios for phosphosites and non-phosphopeptides at the 6-, 12-, 18-, and 60-s time points relative to unstimulated gametocytes (0 s). The non-phosphopeptide ratio distributions were used as null distributions to test significance of the phosphosite ratios. (E) The interquartile ranges (IQRs) of *log*_2_ ratio distributions from (D), with least-squares regression lines and Pearson correlation coefficients shown. (F) Hierarchical clustering of *log*_2_ ratios of the significantly regulated phosphosites.

Activation of gametocytes by XA at a permissive temperature is very rapid (Figure 1A). To capture the cellular response to the initial Ca^2+^ signal, we focused our time course on the first 18 s, at 6-s intervals. The time course finishes after the first minute, since this period should be sufficient to capture the key events associated with the induction of major cell cycle events in the microgametocyte. Parasite samples were mixed-sex populations, so we could not, therefore, isolate microgametocyte-specific events. However, known sex-specific expression patterns of genes and proteins allow many phosphorylation events to be interpreted in the context of sex-specific biology (Khan et al., 2005, Talman et al., 2014).

For each time point, we used four label-free biological replicates, and we produced two technical replicates from each phosphopeptide-enriched sample (Figure 1B). A total of 17,238 unique peptides was identified, of which 8,982 were phosphopeptides; 12,672 phosphosites were identified within these peptides, with 10,237 localized at site level with high confidence (confidence *>* 0.75); and 8,299 of these high-confidence sites were found on *P. berghei* proteins, while 1,938 were from *Mus musculus* proteins (Table S1). Phosphosite intensities, estimated from the intensities of the different phosphopeptides carrying the sites, were strongly correlated among replicates (Figure 1C). The data cover several of the proteins and phosphosites observed in a previous, 2D gel electrophoretic screen of phosphorylation in gametocytes and gametes (Alonso-Morales et al., 2015). Thus, the data represent a comprehensive and reproducible view of early phosphorylation during gametogenesis, from which we could reliably reconstruct time courses.

### Phosphorylation Time Courses Cluster into Distinct Response Groups

We quantified the change in phosphorylation states over time by calculating *log*_2_ ratios against unstimulated gametocytes (0 s), producing time courses of changes in abundance from 0 to 60 s. To account for sites that were not detectable as phosphorylated at the beginning or end of the experiment, we also constructed two truncated time courses: 6- to 60-s time courses for sites that were not observed at 0 s and 0- to 18-s time courses for those that were not observed at 60 s.

1,089 phosphosites on 549 *P. berghei* proteins showed evidence of significant change compared to non-phosphopeptides (Figure 1D), referred to herein as regulated sites; however, it is important to emphasize that this is in reference to regulation of phosphorylation state and may not, in all cases, correspond to regulation of protein activity. The distributions of fold changes were found to widen over time, meaning that more sites were significantly regulated as the activation process progressed (Figure 1E). This has been interpreted as the signaling response spreading out to a wider range of targets over the course of the experiment (Kanshin et al., 2015). For most regulated sites, full, 0- to 60-s time courses were measurable (0–60 s, 926 sites; 6–60 s, only 64 sites; 0–18 s, only 99 sites; Table S2). Hierarchical clustering of the time courses revealed general upregulation trends for the majority of the sites and downregulation for only about a fifth of the sites (Figure 1F).

**Figure 2:**
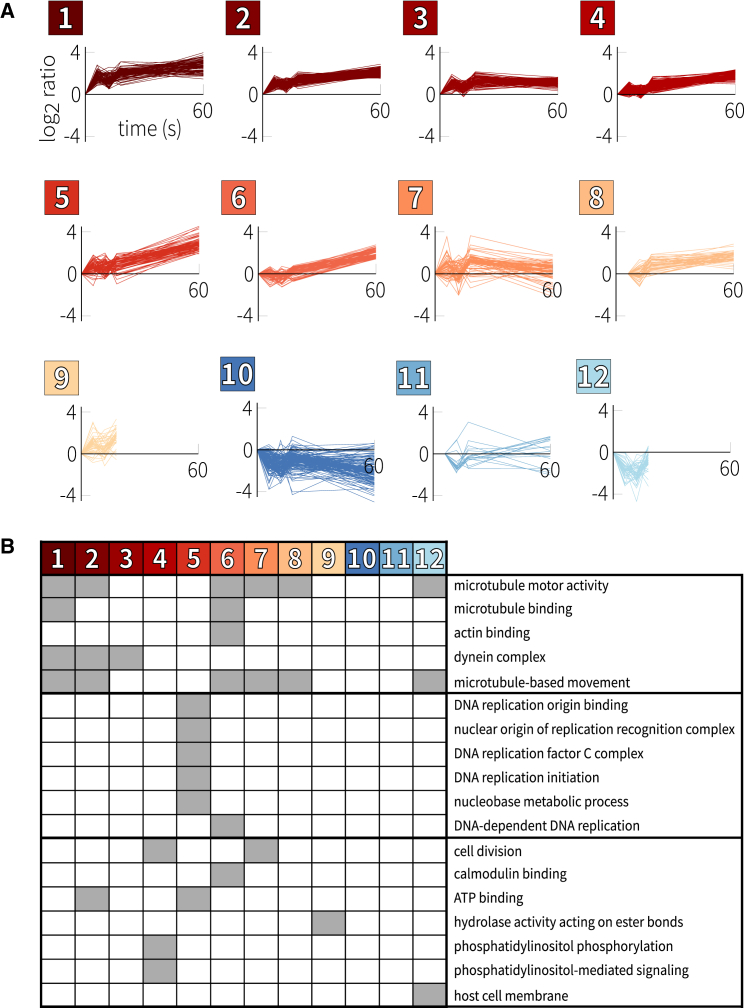
Phosphosite Clustering (A) Gaussian mixture-model-based clustering of *P. berghei* phosphorylation time courses. (B) Enriched gene ontology terms for the *P. berghei* time course clusters. See also Figure S1.

To gain finer detail, we employed a Gaussian mixture-model clustering algorithm to re-cluster the data (Figure 2A; see also Table S2). This method has the advantage of heuristically choosing the optimum number of clusters based on a likelihood measurement. The 0- to 60-s time courses were thus grouped into eight clusters: seven that showed general upregulation and one that showed downregulation. Clusters 1, 2, and 3 consist of sites that were already upregulated within 6–12 s, while clusters 4, 5, and 6 showed predominantly later upregulation. Cluster 7 contains upregulated sites that did not fit well in the other clusters. For the truncated time courses, the algorithm produced two clusters each (one upregulated and one downregulated) (Figure 2A; 6–18 s, clusters 8 and 11; and 0–18 s, clusters 9 and 12). We found that the phosphosites in the parasite were characterized by several classes of dynamic responses to gametocyte activation, with many displaying activity within the first seconds after the initial stimulus.

### DNA Replication- and Microtubule-Related Proteins Are Primary Targets of Phosphoregulation

Of the 549 significantly regulated proteins, 436 were annotated with at least one gene ontology (GO) term, which we used to detect biological functions enriched within individual phosphosite clusters (Figure 2B; see Figure S1 for more detail and Table S3 for test statistics; all significant terms have p < 0.05 after correcting for multiple testing). This analysis was consistent with the known importance of cell cycle events during male gametogenesis. Microtubule-related terms were associated with clusters of diverse phosphorylation kinetics during the first minute after gametocyte activation (compare for instance clusters 1 and 6 in Figure 2A, which are both enriched in proteins annotated for microtubule motor activity), potentially reflecting different roles in spindle and axoneme assembly. Proteins functioning in DNA replication initiation and nucleic acid metabolism, on the other hand, were only moderately phosphorylated within the first 18 s (cluster 5), and their phosphorylation continued to increase after 18 s (clusters 5 and 6).

**Figure 3:**
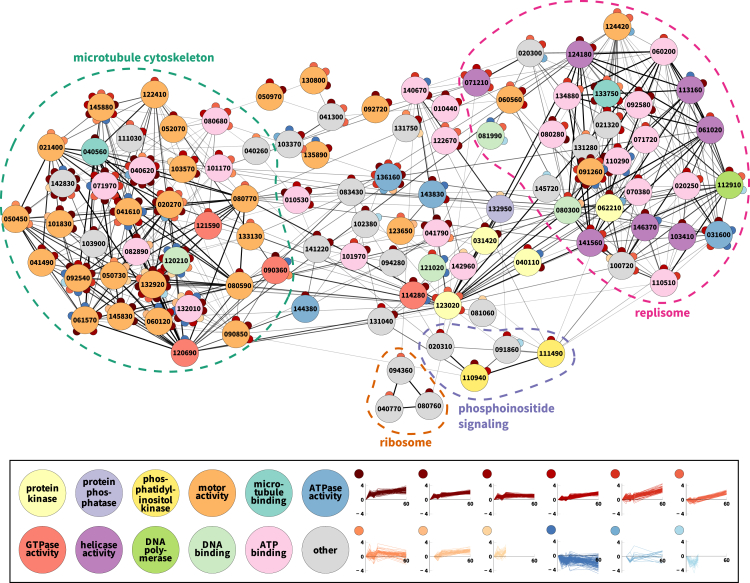
Association Analysis of Sites and Proteins Regulated during Gametocyte Activation Shown is the subset of the functional association network consisting of regulated phosphoproteins that were associated with enriched GO terms. Regulated proteins have evidence of tightly interconnected functional associations. See also Figure S2. Edges (associations) are shaded according to their score (minimum score of 300). Nodes (proteins) are colored according to molecular function annotations and contain the numeric portion of the proteins’ IDs (e.g., “123020” implies “PBANKA_123020”). Small circles represent significantly regulated phosphosites on each protein, with their color reflecting their time course cluster.

When considering the phosphoregulated proteins in the context of a network of functional protein associations, we found that they are more highly interconnected in their associations than expected by chance (Figure S2). We then considered the network of only the regulated proteins that were also associated with enriched functional terms (Figure 3). The visualization shows two major clusters. One is composed of proteins annotated for microtubule motor activity and that tend to be phosphoregulated at many different sites. About a third of these have previously been implicated as components of the axoneme (Talman et al., 2014). The other major cluster consists largely of helicases, protein kinases, and DNA-binding proteins, comprising the replisome. Thus, not only does phosphorylation particularly occur on microtubule- and replication-related proteins but also these proteins show strong evidence for functional association between each other. Phosphoregulation of multiple cellular processes is thus tightly coordinated during gametocyte activation.

Sex-specific proteomic data suggest 125 regulated proteins are male, 65 female, and 129 shared (Tao et al., 2014). One early and one late upregulation cluster (clusters 2 and 6) were enriched in male-specific proteins (60 proteins, p = 0.035; and 37 proteins, p = 0.023, respectively), while another early upregulation cluster (cluster 3) was enriched in female-specific proteins (29 proteins, p < 0.001). The primary downregulation cluster (cluster 10) was enriched in proteins that are found in both sexes (51 proteins, p = 0.035). Although the sex specificity is known for only a subset of the proteome, given the functional enrichment results and known biology of gametogenesis, we suspect that the observed phosphorylation is dominated by microgametocyte activity.

Our global phosphorylation analysis was in good agreement with our recent identification of direct CDPK4 substrates (Fang et al., 2017). Microgametocytes enter S-phase I within 30 s of activation, and, accordingly, CDPK4 and two of its known substrates carry sites that are upregulated early (cluster 3). In both cases, this included the exact residues targeted directly by CDPK4, i.e., S9 in PBANKA_072020 (SOC1) and S5051 in PBANKA_144220 (SOC2). Interestingly, the biological functions associated with these substrates are quite different, with a knockout of SOC1 indicating a role in the initiation of replication, while SOC2 was crucial to assemble the mitotic spindle during microgametogenesis (Fang et al., 2017). SOC2 has additional phosphosites upregulated later (clusters 6 and 7), and it may thus integrate multiple signals from different kinases to regulate the stability of the mitotic spindle. In other eukaryotes, microtubules are nucleated from a ring of γ-tubulin in complex with a family of interacting proteins, many of which are phosphorylated by mitotic protein kinases (Teixidó-Travesa et al., 2012). The only conserved component of the *Plasmodium* ring complex in addition to γ-tubulin, PBANKA_083430, is characterized in our data by an early phosphorylation event from 6 s, as are the centrosome component centrin-2, a number of axonemal dynein chains, and other axoneme-associated proteins (Talman et al., 2014) in the rapid response clusters 1–3.

Phosphatidylinositol (PI) signaling is known to be triggered early by PKG and is required for the rapid mobilization of Ca^2+^ (Brochet et al., 2014). We found regulated phosphosites on a PI 3-kinase (PBANKA_111490), a PI 4-phosphate-5-kinase (PBANKA_020310), and a PI 4-kinase (PI4K; PBANKA_110940) that is a validated drug target (McNamara et al., 2013). PI metabolism was enriched in a late upregulation cluster (cluster 4), indicative of roles in signal termination or restoration of IP3 levels. However, closer inspection of the data revealed two regulated sites on PI4K, S534 (cluster 2) and S538 (cluster 4), which both responded at 6 s, consistent with the early function of PI4K in generating the gametocyte Ca^2+^ signal, as proposed by Brochet et al. (2014). S538 showed a second peak at 60 s, hence its inclusion in the late upregulation cluster. Interestingly, the same sites are direct or indirect targets for PKG in ookinetes, where they are functionally important for gliding (Brochet et al., 2014).

Taken together, these results indicate that our data cover most of the few phosphorylation events known to be involved in the regulation of the *Plasmodium* cell cycle and should thus contain other functionally relevant events. The functional enrichment analysis highlights high-level trends that are congruent with our limited existing knowledge of gametogenesis, and it further indicates that DNA replication and mitosis may be initiated simultaneously and not sequentially as the classical cell cycle model would suggest.

### Protein Kinases and Phosphatases Associate with Specific Dynamic Clusters

The cell cycle in the sexual stages is likely to be orchestrated by both stage-specific and more constitutively expressed regulators, many of the latter being intractable by non-conditional gene knockout. The presence of such protein kinases among the phosphoregulated proteins could potentially reveal otherwise difficult-to-obtain functional information through the time course data. By using, on the one hand, the protein-protein association network to infer functional relationships between proteins and, on the other hand, our phosphosite clusters to identify groups of proteins undergoing phosphoregulation with similar dynamics, we can statistically test whether the kinases are significantly associated with any of these regulated protein groups. Briefly, we tested whether the median association score (MAS) between a kinase and a group of regulated proteins is higher than that of all the kinase’s associations.

**Figure 4:**
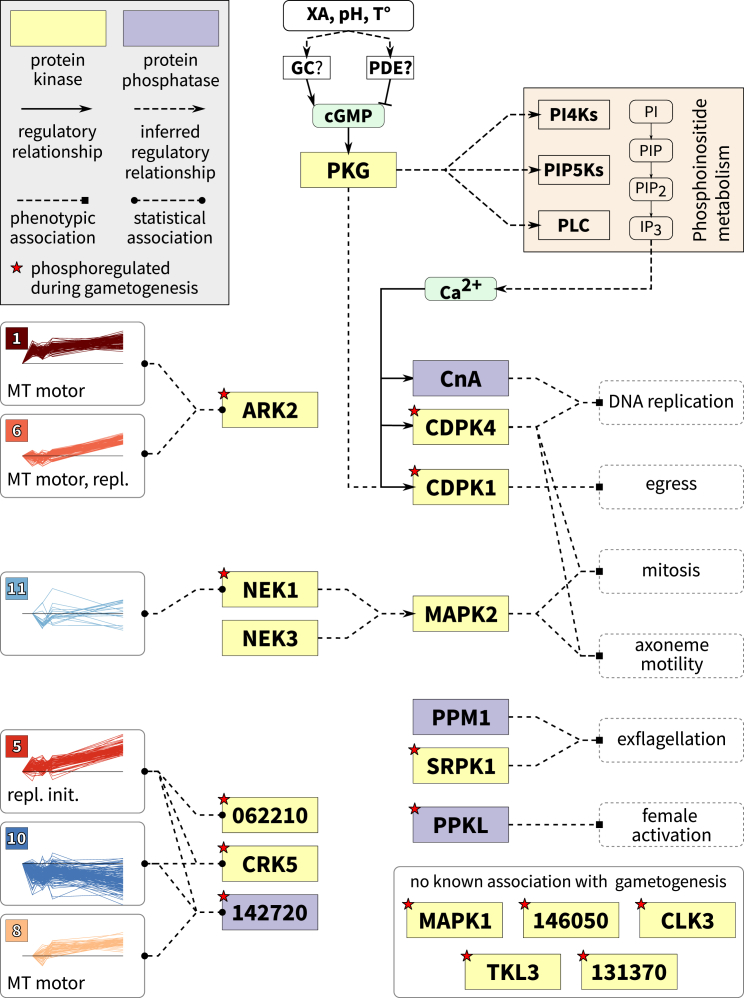
A Schematic of Early Phosphorylation Signaling during Gamete Formation Some enzymes can be associated with the phosphosite clusters via statistical enrichment on a protein-protein functional association network; enriched functional terms for each cluster are listed (MT motor, microtubule motor activity; repl. init., replication initiation; and repl., DNA replication). Other enzymes have been associated with specific stages of gametogenesis through genetic manipulation. Inferred regulatory relationships include cases in which specific phosphoregulation of unknown function occurs.

For example, an aurora kinase, ARK2 (PBANKA_040740; 252 associations, MAS = 276), had two upregulated phosphosites. It had significant associations with proteins with rapidly phosphorylated sites (cluster 1) or late-phosphorylated sites (cluster 6) (for both cases, n = 7, MAS = 590, p = 0.042; Figure 4). Both phosphosite clusters were enriched in microtubule motor proteins, many of which were also functionally associated with ARK2, with little overlap between the two sets of proteins. This links ARK2 to the phosphoregulation of the microtubule motor proteins, which is consistent with another member of the aurora kinase family being found at microtubule-organizing centers in *P. falciparum* (Carvalho et al., 2013).

The essential NIMA-related kinase NEK1 (PBANKA_144300; 787 associations, MAS = 216) is thought to be involved in MAPK signaling (Khan et al., 2005, Dorin-Semblat et al., 2011, Carvalho et al., 2013). Its single phosphoregulated site was rapidly dephosphorylated within 6 s and recovered to pre-activation levels by 18 s. On the network, it associated with similarly regulated proteins (cluster 11) (n = 3, MAS = 349, p = 0.048; Figure 4).

In a more complicated case, we found overlapping patterns of functional associations between CRK5 (PBANKA_123020; 1,055 associations, MAS = 281), the orphan kinase PBANKA_062210 (1,247 associations, MAS = 244), and a putative protein phosphatase 2C (PBANKA_142720; 554 associations, MAS = 237.5). CRK5 was upregulated at multiple sites following gametocyte activation, while PBANKA_062210 and the phosphatase had similar patterns of up- and downregulation at two sites. All three enzymes were associated with proteins that were predominantly upregulated at later time points, with some early activity (cluster 5), linking the enzymes with DNA replication initiation proteins (CRK5: n = 20, MAS = 397.5, p = 0.048; PBANKA_062210: n = 32, MAS = 384, p < 0.001; and PBANKA_142720: n = 4, MAS = 712, p = 0.028; Figure 4). CRK5 and the phosphatase also had significant interactions with proteins undergoing broad dephosphorylation (cluster 10) (CRK5: n = 37, MAS = 395, p = 0.048; PBANKA_142720: n = 12, MAS = 440, p = 0.028). Lastly, the phosphatase was associated with proteins that underwent late upregulation (cluster 8) (n = 7, MAS = 430, p = 0.034), which was enriched in microtubule motor proteins. Such inter-enzyme associations further highlight the probability that we have observed a single, broadly functioning signaling module that coordinates cell cycle progression in gametocytes.

For completeness, Figure 4 incorporates published data from *P. falciparum*, identifying CDPK1 as a substrate of PKG (Alam et al., 2015) and MAPK2 as an *in vitro* substrate of NEK1 and NEK3, suggesting that they could regulate the functionality of this kinase (Lye et al., 2006, Dorin-Semblat et al., 2011). None of the previously implicated phospho-enzymes were otherwise found to be statistically associated with the time course clusters. This is likely due to low coverage of the network, in which many of the enzymes, such as CDPK4 and SRPK1, have relatively few high-confidence associations. Nevertheless, the results introduce several new enzymes as likely components of the pathway, with their specific roles requiring further validation.

### Conservation of Phosphosites Suggests Functional Constraint

If a phosphosite has a vital biological function, it is likely to be conserved at the sequence level, and phosphorylation at homologous sites should be observable in different species. We assessed the sequence conservation of phosphosites at the underlying nucleotide level (*dN*/*dS*) per site during *Plasmodium* evolution (Table S4). We defined conserved sites as those that show evidence of more selective constraint (lower *dN*/*dS*) than would be predicted by known determinants of evolutionary rates, such as local protein disorder or overall gene expression levels. The proteins with conserved phosphosites were enriched for motor activity, the myosin complex, calmodulin binding, and cytoskeletal protein binding. When limited to only sites undergoing active phosphoregulation during gametogenesis, the set was enriched in proteins annotated for DNA helicase activity, DNA replication and replication initiation, and the MCM complex.

We then focused on the conserved, regulated sites that fall within predicted protein domains and have previously been observed as phosphorylated in *P. falciparum*. Together, these would be likely indicators of important functionality. We identified 13 such sites (Table 1). Notably, both CDPK1 and CDPK4 each have one such phosphosite located within their kinase domains, suggesting that these sites can be taken as indicators of the respective kinase’s activity. This is supported by the fact that the orthologous CDPK1 site in *P. falciparum* has been identified as a site of autophosphorylation (Ahmed et al., 2012). Several sites also fall within domains related to DNA replication and the cell cycle, for which evidence in other species suggests some functionality. In particular, phosphorylation within the MCM N-terminal domain of human MCM7 has been implicated in the regulation of formation of the MCM2-7 complex and in the progression of the cell cycle (Wei et al., 2013), and phosphorylation within the MCM domain of human MCM3 regulates the replication process (Han et al., 2015). Overall, these patterns of conservation indicate that phosphorylation likely plays an important role in the physiological regulation of these proteins.

**Table 1:** Conserved Phosphosites

Protein ID	Description	Site	Domain	*dN*/*dS*
PBANKA_020300	chromatin assembly factor 1 protein WD40 domain	S50	CAF1C H4-bd (PF12265.5)	0.011
PBANKA_031420	CDPK1	S65	protein kinase domain (PF00069.20)	0.05
PBANKA_041680	ubiquitin specific protease	S1076 / S1077	ubiquitin carboxyl-terminal hydrolase (PF00443.24)	0.012 / 0.049
PBANKA_051190	RPL3	S13	ribosomal L3 (PF00297.19)	0.013
PBANKA_061090	HSP40, subfamily A	S181	DnaJ central domain (PF00684.14)	0.008
PBANKA_061520	CDPK4	S80	protein kinase domain (PF00069.20)	0.003
PBANKA_071590	USP13	Y410	UCH (PF00443.26)	0.02
PBANKA_080310	MCM7	T154	MCM N-terminal domain (PF14551.1)	0.006
PBANKA_081570	transporter	S251	Major Facilitator Superfamily (PF07690.11)	0.024
PBANKA_111190	MAT1	T94	MAT1 (PF06391.10)	0.015
PBANKA_112250	conserved *Plasmodium* protein, unknown function	Y108	SF1-HH (PF16275.2)	0.018
PBANKA_124180	MCM3	S626	MCM (PF00493.20)	0.007

These significantly regulated sites, which show strong conservation, lie within a predicted protein domain, and have been previously observed in *P. falciparum*, are predicted to have functional importance. See also Figure S3.

We were also intrigued by the large number of phosphoregulated sites on individual motor proteins. We identified phosphorylated regions of putative functional importance in these protein families via a phosphorylation hotspot analysis (Beltrao et al., 2012) (Figure S3). A regulated phosphosite on a dynein light intermediate chain (PBANKA_041490, T629) fell in one such conserved hotspot (Figure S3A, alignment position 850). Although the site was predicted to be in a disordered region, and thus more phosphorylation activity was expected around it, the hotspot was well aligned and fell within the predicted dynein light intermediate chain domain. The orthologs in humans (Uniprot: O43237) and mice (Q6PDL0) are also phosphorylated at this position (S383 in both species), albeit with no known function. In the dynein intermediate chain family, an N-terminal hotspot has a known regulatory role (see, e.g., Vaughan et al., 2001). We found one regulated site nearby on PBANKA_050450 (S120; Figure S3B, alignment position 232); while not in a structured region, its proximity to a hotspot of known functional importance in other members of the protein family merits further investigation. Finally, the dynein heavy chain and kinesin families were found to be heavily and evenly phosphorylated along their full lengths, indicating that these proteins are frequent targets of phosphorylation in eukaryotes (Figures S3C and S3D). Overall, our observations of motor-protein phosphoregulation are consistent with broad phosphorylation patterns observed across their protein families between highly divergent species, and they suggest conserved modes of functional regulation.

### Gene Knockouts Link Protein Kinases to Core Signaling Responses

We next investigated the roles of specific protein kinases in the gametocyte activation response. We studied two knockout lines, one lacking CDPK4 and the other SRPK1. Both mutants make morphologically normal gametocytes but are defective in male gametogenesis (Billker et al., 2004, Tewari et al., 2010). While CDPK4 has been the subject of recent targeted analysis (Fang et al., 2017), much less is known about the specific activities of SRPK1. Both kinases undergo phosphoregulation during our time course, but they showed poor connectivity on the network. We thus employed the knockout lines to compare phosphorylation patterns before and 18 s after activation, with and without each kinase. We confirmed the reliability of the results by comparing to known functionality for CDPK4, and then we focused on new observations in SRPK1.

In the CDPK4 knockout experiment (CDPK4-KO), we quantified 3,539 unique peptides in the wild-type (WT) and knockout (KO) mixed-sex samples, belonging to 993 *P. berghei* proteins and 663 *M. musculus* proteins (Table S5). In the SRPK1-KO experiment, 3,949 peptides were quantified from 1,060 *P. berghei* proteins and 787 host proteins (Table S6). Of the 1,089 significantly regulated phosphosites from the time course experiment, only 190 were unambiguously quantified in CDPK4-KO and 236 in SRPK1-KO. The small overlap between experiments can be accounted for by the inherent stochasticity of mass spectrometry (MS) peptide detection in complex samples, combined with the fact that only two time points were taken for the mutants.

**Figure 5:**
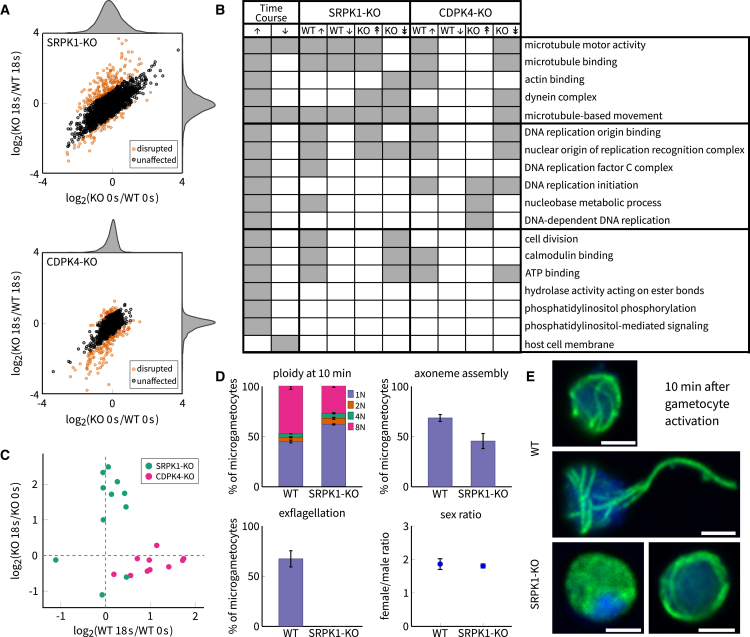
Disruptions of CDPK4 and SRPK1 Produce Distinct Effects on the Phosphorylation Response during Early Gametogenesis (A) *log*_2_-fold changes of phosphosite abundance from 0 to 18 s compared between mutant and wild-type gametocytes (plus marginal density plots). Peptides whose phosphoregulation is specifically altered during gametogenesis in the mutant are shown in orange. (B) A comparison of GO term enrichment results for the time course experiment and the two KO experiments (WT, up/down-phosphoregulation; KO, positive/negative disruption). Gray squares indicate significant enrichment. (C) Disrupted phosphopeptides from motor proteins in the two KO experiments. Without SRPK1, several motor proteins become upregulated during gametogenesis, while lack of CDPK4 results in the loss of phosphorylation events. (D) The effects of knocking out SRPK1 on replication and exflagellation (2 replicates; data are represented as mean ± SEM). (E) Confocal microscopy images of WT (top, middle) and SRPK1-KO (bottom) parasites 10 min after activation. By this time, WT is initiating (top) or undergoing (middle) exflagellation, while some SRPK1-KO have exhibited no DNA replication or axoneme formation (bottom left), or replication and axoneme formation but no exflagellation (bottom right). Gametocytes were immunofluorescence-labeled for mouse anti-α-tubulin antibodies with anti-Mouse Alexa488 as a secondary antibody (green). DNA was counterstained with DAPI (blue). Scale bars, 2 μm.

Pre-activation differences between KO and WT may have been caused by accumulated perturbations to protein abundances or phosphorylation during gametocyte formation, which would persist during gametogenesis if not under further regulation by the kinase. Indeed, for both experiments, we found a positive correlation between pre- and post-activation disruptions (Figure 5A). We interpreted outliers to this correlation as being specifically dependent on the kinase during gametocyte activation (Figure 5A, orange points). Of 196 CDPK4-dependent phosphosites, most were negatively disrupted in the KO. In contrast, of 243 SRPK1-dependent sites, the majority were positively disrupted. Thus, deleting CDPK4 leads to a significant loss of phosphorylation events, while a lack of SRPK1 produces notable gains thereof as well as a loss of dephosphorylation events. This suggests SRPK1 may indirectly repress phosphorylation via an intermediary enzyme.

A functional enrichment analysis on the disrupted proteins (Figure 5B) reassuringly showed that WT samples largely replicated the time course experiment. The KO samples were enriched for microtubule cytoskeleton in negatively disrupted proteins in CDPK4-KO and in both positively and negatively disrupted proteins in SRPK1-KO. Specifically, in the case of CDPK4 the phosphosites were upregulated in WT and unregulated in the KO after activation; while for SRPK1 the trend was reversed (Figure 5C). Notably, the specific phosphosites that were disrupted did not overlap between mutants, suggesting that the two protein kinases have different sets of substrates. We also saw a disruption in both experiments of DNA replication origin binding, through components of the origin of replication initiation complex (ORC) and the MCM complex (Figure 5B). An interesting case is S60 on ORC1 (PBANKA_060200), which was upregulated after activation in WT. When CDPK4 was not present, regulation of this site was lost. However, when SRPK1 was not present, the site significantly increased in abundance relative to WT. This suggests that the signal for phosphorylation of this site originates with CDPK4 and the stoichiometry of this event is moderated by the signal passing through an SRPK1-mediated feedback loop. This is supported by the observation that phosphorylation of SRPK1 itself was negatively disrupted when CDPK4 was deleted, which would place SRPK1 downstream of CDPK4 in the early gametogenesis-signaling events. This, however, complicates the interpretation of the independent disruptions of motor proteins.

The phosphoproteomic effects of deleting CDPK4 fit our general understanding of the role of this kinase following gametocyte activation and thus impart confidence in the SRPK1-KO results. To place the SRPK1-KO phosphoproteomic results in a broader phenotypic context, we measured DNA replication, axoneme assembly, and exflagellation of the mutant parasites. We found that, by 10 min, a significantly lower percentage of gametocytes had completed three rounds of DNA replication, while a larger percentage remained haploid (Figure 5D). SRPK1-KO male gametocytes thus appear affected during the first genome replication, but, if this process completes, no further defects are observed in reaching the octoploid level. Consistently, we observed that parasites that reached the octoploid level successfully assembled axonemes (Figures 5D and 5E). While neither observation could be accounted for by a change in sex ratio in the mutant parasites (Figure 5D), they may be due to some proportion of the microgametocyte population becoming nonviable during gametocytogenesis. This is tentatively supported by our phosphoproteomic results, which showed marked differences in the SRPK1-KO already at pre-activation. However, the data also revealed significant post-activation effects on phosphorylation, which may account for the observed phenotype. Thus, the kinase is expected to play a regulatory role in the seconds following activation. Furthermore, since microgametocytes lacking SRPK1 completely failed to exflagellate (Figure 5D; see also Tewari et al., 2010), this kinase is additionally required for fully replicated cells to complete gametogenesis.

## Discussion

*Plasmodium* gametocytes must detect the change in environment from host to vector and propagate that signal quickly and efficiently to initiate all constituent events of gametogenesis. Because this is vital to the parasite’s successful transmission, determining the underlying signaling events is a primary step toward a fundamental understanding of transmission. Our results reveal a prominent phosphorylation response within 18 s after gametocyte activation and that this signal is widespread by 60 s. In particular, despite having observed a large number of proteins undergoing active phosphoregulation during the response in mixed-sex samples, we found a large fraction of the activity to occur on proteins pertaining to DNA replication and mitosis, two processes known to be rapidly induced in the male gametocyte upon activation. Interestingly, we also observed phosphorylation events on female-specific proteins during the same period; however, it is less clear which processes might be undergoing regulation in the macrogametocytes.

We observed phosphoregulation on several protein kinases and phosphatases, many of which are dispensable for the asexual erythrocytic cell cycle but that were implicated through gene-KO studies in regulating gametocyte activation specifically, including CDPK1 and CDPK4, MAPK2, SRPK1, CnA, PPM1, and PPKL. In most cases, we cannot yet directly relate phosphorylation state to kinase activity levels. However, because some kinases show significant functional association with other proteins undergoing phosphoregulation, we can infer that they are likely to play important roles. In this manner, we identified ARK2, CRK5, NEK1, and the orphan kinase PBANKA_062210 as putative members of the gametocyte activation-signaling pathway. While no functional data are available on the orphan kinase, the first three candidates all belong to more conserved protein kinase subfamilies implicated in regulating the eukaryotic cell cycle. These have evidence for expression in asexual stages, and attempts to disrupt these genes have been consistently unsuccessful in *P. berghei* (Tewari et al., 2010, Solyakov et al., 2011). Roles for these protein kinases are, therefore, most likely not limited to gametogenesis, but they may extend to regulating the *Plasmodium* cell cycle more generally. Stage-specific or inducible mutants in these genes have the potential to reveal deeper insights into the mechanisms of *Plasmodium* cell cycle regulation.

A comparison of the distinct effects of deleting CDPK4 and SRPK1 highlights the potential for complex modes of regulation in this system. CDPK4 appears to be directly or indirectly responsible for the phosphorylation of many proteins within the first 18 s. Since CDPK4 is known to be activated in the microgametocyte soon after the XA signal is detected, this places this kinase upstream in the pathway with a master regulator role, as previously suggested by Billker et al. (2004). SRPK1, on the other hand, plays a more indirect role, in which its presence is responsible for the repression of phosphorylation. The disruption of replisome and microtubule cytoskeleton protein phosphorylation, with a concomitant reduction of DNA replication and axoneme formation, in the SRPK1 KO mutants implicates SRPK1 in gametocyte cell cycle regulation rather than, or in addition to, its expected role of regulating SR protein-mediated RNA processing. We hypothesize that SRPK1’s activity depends on activation of CDPK4 and that it, in turn, either negatively regulates another kinase or positively regulates a phosphatase. In at least one case, CDPK4 and SRPK1 appear to cooperate in a feedback-like manner in the regulation of a protein. Considered in the light of the parasite’s reduced protein kinase repertoire (76 in *P. berghei*) and the rapidity and efficiency of gametogenesis, this provides an intriguing glimpse at the complexity of the underlying protein signaling.

Cell cycle regulation in malaria parasites is poorly understood, and current evidence suggests that some of the canonical cell cycle checkpoints are not present. During asexual schizogony in the blood stages, for example, nuclei behave as autonomous units that undergo repeated rounds of replication and mitosis in an asynchronous manner, while being retained within the same cellular envelope (Read et al., 1993, Arnot et al., 2011). During sexual development, the release of microgametes requires DNA synthesis (Janse et al., 1986). On the other hand, a compound that interferes with spindle formation did not prevent DNA replication to proceed through all three rounds (Billker et al., 2002), suggesting that in the male nucleus multiple rounds of replication and mitosis may progress in parallel without depending on one another. We were therefore intrigued to see that proteins involved in origin of replication recognition and cytoskeletal reorganization were simultaneously targeted for phosphorylation during the first few seconds of gametocyte activation. These data indicate that some mitotic processes are already initiated contemporaneously with DNA synthesis in *Plasmodium* gametocytes, and they raise important questions regarding the temporal control of the cell cycle in these organisms.

## Experimental Procedures

### Parasite Maintenance and Preparation

Work involving rodents was reviewed by the Animal Welfare and Ethical Review Body of the Wellcome Trust Sanger Institute and licensed by the UK Home Office or according to the guidelines and regulations issued by the Swiss Federal Veterinary Office with authorization GE/82/15. *P. berghei* strain ANKA clone 2.34, CDPK4-KO (Fang et al., 2017), and SRPK1-KO (Tewari et al., 2010) were maintained in CD1 outbred mice. Female mice were specific pathogen free and subjected to regular pathogen monitoring by sentinel screening. Mice were used for experimentation at 6–11 weeks of age. For gametocyte production, mice were treated with phenyl hydrazine 3 days before infection. One day after infection, asexually replicating parasites were eliminated by the addition of sulfadiazine (20 mg/L) in the drinking water. Parasites were harvested at day 4 after infection in suspended animation and separated from uninfected erythrocytes. Activation was induced and parasites were snap-frozen in liquid nitrogen at 6, 12, 18, and 60 s (time course) or at 18 s (KO) after activation. For each time point (including unactivated 0 s) and parasite line, four and two independent biological replicates were produced for the time course experiment and the KO experiments, respectively. See the the Supplemental Experimental Procedures for more details.

### Quantitative Protein MS

#### Time Course Experiment

Cell samples were lysed and the protein content was purified and digested using the filter-aided sample preparation (FASP) method (Wiśniewski et al., 2009). Phosphopeptide enrichment was performed on TiO2 tips (Thermo Fisher). Two technical replicates of each sample were analyzed on an LTQ Orbitrap Velos coupled with an Ultimate 3000 RSLCnano System (both from Thermo Fisher). Raw spectra from the time course experiment were analyzed using MaxQuant (version 1.5.2.8) (Cox and Mann, 2008), treating technical replicates as fractions. Peptides were searched against the *P. berghei* annotated protein database retrieved from PlasmoDB (http://plasmodb.org/plasmo/; version 13.0) and the *M. musculus* protein sequence database retrieved from Uniprot (http://www.uniprot.org). Only phosphosites with a localization probability of 0.75 or greater were retained for further analysis. For full protocol details and analysis parameters, see the Supplemental Experimental Procedures.

Time courses for the change in phosphorylation level for each phosphosite were calculated using the composite intensity scores estimated by MaxQuant from all peptide evidence for that site. Following stringent quality control, three time courses were then generated from these values: the full time course (0–60 s) and two truncated time courses for sites either not detected in unactivated parasites (6–60 s) or at the end of the experiment (0–18 s). For each site, *log*_2_-transformed ratios were calculated from its intensity at each time point against its intensity at the first time point, e.g., *log*_2_(6s/0s). Only complete time courses, with no missing data, were retained for further analysis.

To determine which sites show significant evidence of change in phosphorylation state, we exploited the fact that enrichment of phosphopeptides is not 100% specific and that the time frame in question is too short to see significant variation in protein abundances. Time courses were constructed for non-phosphorylated peptides in the same manner as described above. The empirical cumulative distribution functions (ECDFs) of these *log*_2_ ratios were used as null distributions against which the significance of the phosphosite *log*_2_ ratios could be tested. Sites having at least one time point with a p value less than the critical value of 0.05 were determined to have undergone significant phosphoregulation during the time course. Significant sites were clustered according to their time courses using a normal mixture-modeling-based method. See the Supplemental Experimental Procedures for more details.

#### KO Experiments

Cell samples were lysed and proteins were isolated via methyl tert-butyl ether (MTBE) precipitation (Matyash et al., 2008). The proteins were then digested with trypsin and labeled with TMT 10plex. The labeled peptide mixture was fractionated, and enrichment of phosphopeptides was performed using IMAC with PHOS-Select Iron Affinity Gel (Sigma) then TiO2 tips (Thermo Fisher) sequentially. The enriched samples were subjected to liquid chromatography-tandem MS (LC-MS/MS) analysis on an Orbitrap Fusion Tribrid mass spectrometer coupled with an Ultimate 3000 RSLCnano system. The phosphopeptides enriched via IMAC and TiO2 were analyzed separately. Raw data were processed in Proteome Discoverer 2.1 (Thermo Fisher) using both SequestHT and Mascot search engines against a combined protein database of *P. berghei* and mouse as above. For further details, see the Supplemental Experimental Procedures.

The *log*_2_ ratios were calculated for both biological replicates using the peptides’ scaled abundances for the following comparisons for each replicate: KO 18 s versus KO 0 s, WT 18 s versus WT 0 s, KO 18 s versus WT 18 s, and KO 0 s versus WT 0 s. A final, mean ratio was computed from all of the available evidence. For all ratios, a p value was calculated using the ECDF of the non-phosphopeptides, as described above.

To account for a general change in cellular state before gametocyte activation, we built a linear model to predict the disruption at 18 s from the disruption at 0 s. That is, we aimed to predict *log*_2_(KO18s/WT18s) from *log*_2_(KO0s/WT0s). We reasoned that peptides that were poorly predicted by this model could be assumed to have been affected specifically during the activation process. We thus took the outliers to have been under direct influence of the deleted kinase.

### Statistical Testing

Unless otherwise noted, all statistical tests and regression modeling were performed using R version 3.3.0 (R Core; R Core Team, 2016). The p values were corrected for false discovery rate (FDR) (Benjamini and Hochberg, 1995) and tested against a critical value of 0.05. FDR-adjusted p values are reported. Functional enrichment was performed using the binomial test of significance (Mi et al., 2013) on gene ontology associations for *P. berghei*, supplemented with associations imported by orthology from *P. falciparum*. Enrichment for protein sex specificity was performed using a one-sided Fisher’s exact test on a previously published *P. berghei* sex-partitioning dataset (Khan et al. [2005], as reanalyzed by Tao et al. [2014]). Functional association analyses were performed using the STRING association network for *P. berghei* (version 10; Szklarczyk et al., 2015), supplemented with associations imported from the *P. falciparum* network. Significant associations between phospho-enzymes and groups of proteins were tested using the Mann-Whitney test to compare the MAS between the enzyme and those proteins with the median score for all of the enzyme’s association. Conservation was measured by estimating *dN*/*dS* on nucleotide multiple-sequence alignments (MSAs) of the encoding genes for orthologous groups of up to seven *Plasmodium* species. Phosphorylation hotspots were identified by performing kernel density estimation of phosphorylation counts on amino acid MSAs of seven diverse species. For more details on each test, see the Supplemental Experimental Procedures.

## Author Contributions

O.B., P.B., and J.C. supervised the work. O.B., P.B., J.C., M.B., and B.M.I. designed the experiments. M.B. prepared the parasite cultures and performed the SRPK1-KO phenotyping. M.B., B.M.I., and L.Y. prepared the samples for LC-MS/MS analysis. L.Y. performed the LC-MS/MS analysis. B.M.I. performed the data analyses and prepared the figures. All authors contributed to the manuscript text.

## Data and Software Availability

The accession number for the MS proteomics data reported in this paper is ProteomeXchange: PXD006266.

## Supplemental Information

Document S1. Supplemental Experimental Procedures and Figures S1–S3

Table S1. Time Course LC-MS/MS Abundances, including Phosphosites and Non-phosphopeptides

Table S2. Calculated Fold Changes and Cluster Assignments for Phosphosites during the Time Course

Table S3. Functional Enrichment Analysis Results

Table S4. Site-Specific Conservation Data

Table S5. CDPK4-KO Phosphopeptide Fold Changes

Table S6. SRPK1-KO Phosphopeptide Fold Changes

Document S2. Article plus Supplemental Information
