# Supplementary material for: Sub-minute Phosphoregulation of Cell Cycle Systems during Plasmodium Gamete Formation
Source: Cell Rep. 2017 Nov 14;21(7):2017–29. doi: 10.1016/j.celrep.2017.10.071 (PMC5700370; doi:10.1016/j.celrep.2017.10.071)
Supplement: Document S1. Supplemental Experimental Procedures and Figures S1–S3 [file mmc1.pdf]

Cell Reports, Volume *21*

Supplemental Information

Sub-minute Phosphoregulation of Cell Cycle Systems during *Plasmodium* Gamete Formation

Brandon M. Invergo, Mathieu Brochet, Lu Yu, Jyoti Choudhary, Pedro Beltrao, and Oliver Billker

Supplemental Figures

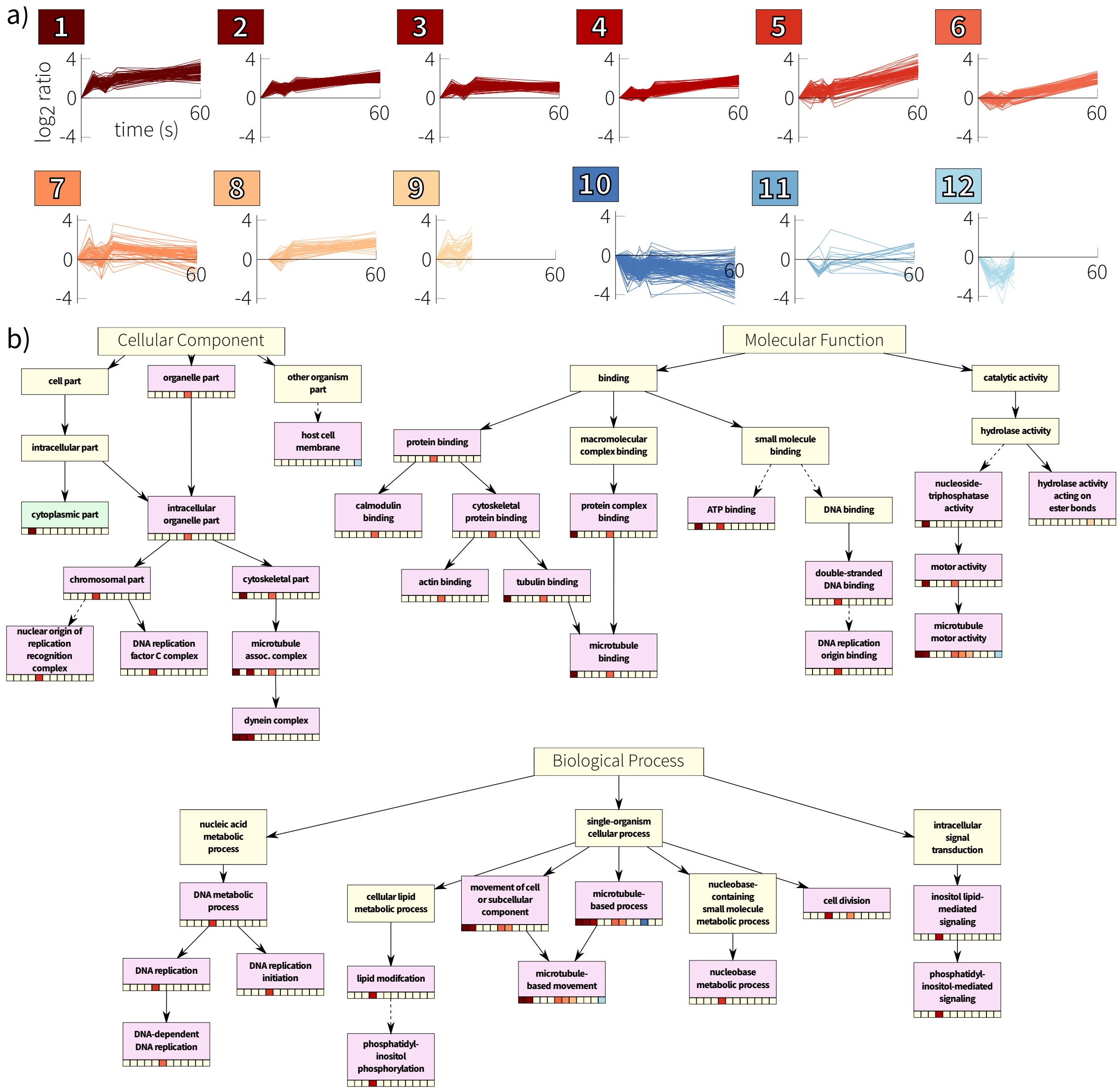

Figure S1: **A detailed view of Gene Ontology enrichment, including higher-level parent terms.** Related to Figure 2. a) Gaussian mixture-model-based clustering of *P. berghei* phosphorylation time courses (identical to Figure 2a, reproduced here for easy reference). b) Enriched (pink) and depleted (green) Gene Ontology terms for the *P. berghei* time-course clusters, including enrichment for more general, parent terms (higher in the graph of terms). For each such term, the clusters enriched/depleted for the term are indicated in the cells beneath the term. The arrangement of the cluster cells, from left to right, matches the numbering in panel (a). Note that some intermediate terms have been removed for clarity; relationships which include removed terms are indicated by dashed lines.

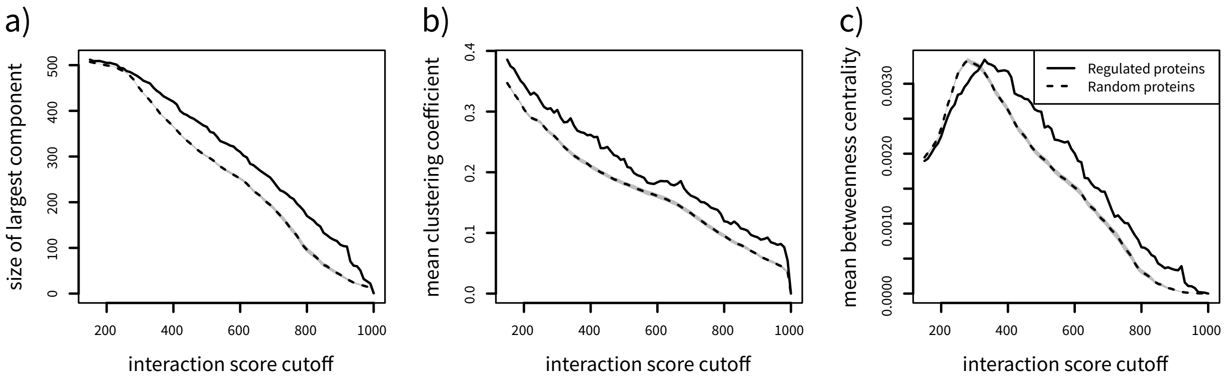

Figure S2: **Phosphoregulated proteins show higher-than-expected interconnectivity.** Related to Figure 3. The subset of the *P. berghei* protein-protein association network that consists only of proteins that were phosphoregulated in the time course experiment shows a) a larger connected component, a) higher median clustering coefficient, and c) higher median betweenness centrality across the full range of interaction score cutoff values than random subsets of the same size. The shaded grey area indicates the 95% confidence interval of the measurements for the random sub-networks.

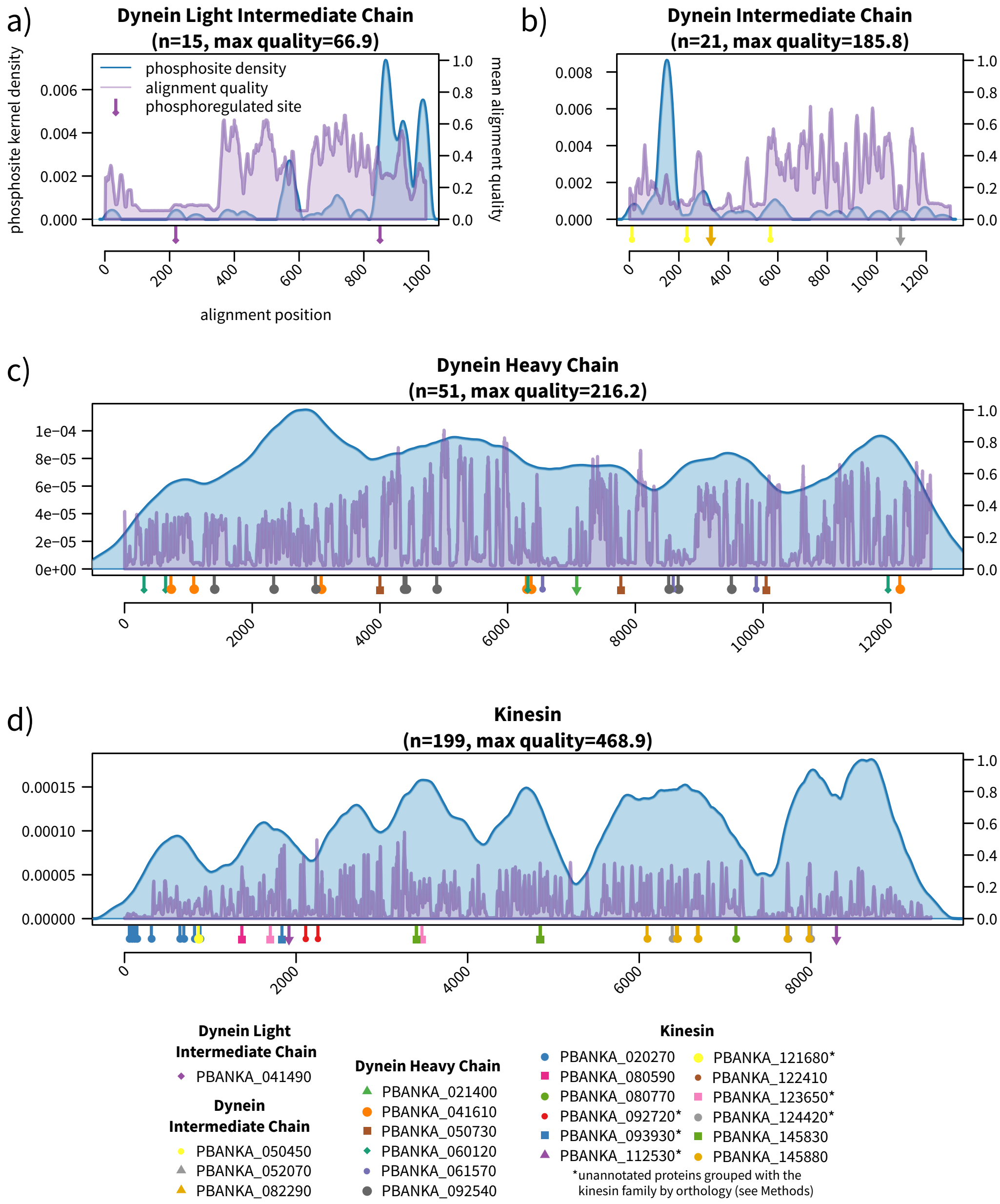

Figure S3: **An illustration of regulated phosphosite positions within conserved phosphorylation hot-spots across motor protein families.** Related to Table 1. Blue traces show the kernel density estimation of phosphosite counts across members of the protein family from *P. berghei*, *P. falciparum*, *T. gondii*, *H. sapiens*, *M. musculus*, *R. norvegicus*, and *S. cerevisiae*. Peaks reflect hot-spots, or regions with a high density of phosphorylation across the protein family. Violet traces show a running mean of the normalised alignment quality. The pins beneath the plots show the locations of *P. berghei* sites that were observed to be under phosphoregulation during gametocyte activation. Dynein light intermediate (a) and intermediate chains (b) have regulated phosphosites at or near phosphorylation hot-spots. Dynein heavy chain (c) and kinesin (d) show broad phosphorylation across their entire lengths, with regulated sites being relatively evenly.

Supplemental Methods

Parasite maintenance and preparation

All animal experiments were conducted under a license from the UK Home Office in accordance with national and European animal welfare guidelines or with the authorisation number (GE/82/15) according to the guidelines and regulations issued by the Swiss Federal Veterinary Office. *P. berghei* strain ANKA (Vincke et al., 1966) derived clone 2.34 (Billker et al., 2004), CDPK4-KO (Fang et al., 2017), and SRPK1-KO (Tewari et al., 2010) were maintained in CD1 outbred mice obtained from Harlan or Charles River. Female mice were specific pathogen-free and subjected to regular pathogen monitoring by sentinel screening. They were housed in individually ventilated cages furnished with a cardboard fun tunnel and Nestlet. Mice were maintained at 21 ± 2 °C under a 12 h light/dark cycle and given commercially prepared, autoclaved dry rodent diet and water *ad libitum*. Mice were used for experimentation at 6–11 weeks of age.

For gametocyte production, mice were treated with phenyl hydrazine three days before infection. One day after infection asexually replicating parasites were eliminated by the addition of sulfadiazine (20 mg/L) in the drinking water. Parasites were harvested at day four after infection in suspended animation (SA - RPMI1640 medium containing25 mM HEPES, 5 % FCS, 4 mM sodium bicarbonate, pH 7.2) and separated from uninfected erythrocytes on a Histodenz cushion made up from 48 % of a Histodenz stock (27.6 % w/v Histodenz -Sigma- in 5.0 mM Tris-HCl [pH 7.2], 3.0 mM KCl, 0.3 mM EDTA) and 52 % SA with a final pH of 7.2. Purified gametocytes were washed twice in SA without FCS and resuspended in 400 µL of SA without FCS. Activation was induced by adding 400 µL of exflagellation medium (RPMI 1640 containing 25 mM HEPES, 4 mM sodium bicarbonate, 200 µM xanthurenic acid, pH 8). Parasites were snap frozen in liquid nitrogen at 6, 12, 18 and 60 s after activation. For the initial time point, the exflagellation medium was replaced by SA. For each time point and parasite line, four and two independent biological replicates were produced for the time-course experiment and the KO experiments, respectively.

A lysis buffer was prepared consisting of 4 % SDS, 50 mM NaCl, 100 mM Tris buffer (pH 7.4), 5 mM EDTA, 40 mM TCEP, and Halt™ Protease & Phosphatase Inhibitor Cocktail (Thermo) (2 x). Samples were split in half (approximately 400 µL each) and 500 µL lysis buffer was added to each half. The samples were vortexed and then heated at 95 °C for 10 min. DNA was sheared via pulses of sonication for 20 s (1 s on, 1 s off) at 40 % power. The samples were centrifuged for 30 min at 14 000 rpm and the supernatant was collected. The samples were then incubated with 80 mM iodoacetamide for 1 h at room temperature. The protein digest used the FASP method (Wiśniewski et al., 2009) with Amicon Ultra-15 30K filter units (UFC903024), where Trypsin Gold (Thermo) was added at a 1:50 ratio and the digest was incubated at 37 °C overnight. The resulting peptides were desalted using Sep-Pak C18 Plus Light cartridges then dried in a SpeedVac and stored at −20 °C.

Quantitative protein mass spectrometry

Time course experiment

Phosphopeptide enrichment Phosphopeptide enrichment was performed on TiO_2 tips (Thermo), following the manufacturer's instructions. Each sample used 500 µg peptides. Phosphopeptides were eluted from the tip by 1.5 % NH_4OH followed by 5 % pyrrolidine. Both eluates were pooled, acidified and then desalted on Graphite Spin Columns (Thermo) as instructed by the manufacturer's protocol. Each sample was split into two technical replicates, dried in a SpeedVac, and stored at −20 °C.

Label-free LC-MS/MS The dried phosphopeptides were resuspended in 80 µL of 0.5 % FA / 100 % H_2O before LC-MS/MS analysis on an LTQ Orbitrap Velos coupled with an Ultimate 3000 RSLCnano System (both from Thermo Fisher). The peptides were first loaded and desalted on a PepMap C18 trap column (100 µm id x 20 mm, 5 µm) then separated on a PepMap C18 analytical column (75 µm id x 500 mm, 2 µm) (both from Thermo Fisher) over a 240 min linear gradient of 4–30 % CH_3CN/0.1 % formic acid with the total cycle time at 280 min. The Orbitrap mass spectrometer was operated in the standard "top 15" data-dependent acquisition mode while the preview mode was disabled. The MS full scan was set at m/z 380–1600 with the resolution at 30 000 at m/z 400 and a lock mass at m/z 445.120 025. The AGC was set at 1×10^6 with a maximum injection time at 200 ms. The 15 most abundant multiply-charged precursor ions, with minimal signals above 3000 counts, were dynamically selected for CID fragmentation (MS/MS) in the ion trap, which had the AGC set at 5000 with the maximum injection time at 100 ms. The dynamic exclusion duration time was set for 60 s with ±10 ppm exclusion mass width. The isolation width was 2.0 Da and the normalised collision energy was 35 %. LC-MS/MS analyses of technical replicates were repeated sequentially.

Spectral analysis and peptide quantification Raw spectra from the time-course experiment were analysed using MaxQuant (version 1.5.2.8) (Cox and Mann, 2008). Technical replicates were treated as fractions. The minimum peptide length was set at seven and protein and site identification false discovery rates (FDR) were set at 0.01. Carbamidomethyl was set as a fixed modification. Peptides were searched against the *P. berghei* annotated protein database retrieved from PlasmoDB (`http://www.plasmodb.org`; version 13.0) and the *M. musculus* protein sequence database retrieved from Uniprot (`http://www.uniprot.org`). Peptides were quantified using the iBAQ (intensity-based absolute quantification) method (Schwanhäusser et al., 2011), as implemented in MaxQuant, using a logarithmic fit and spectra-matching between runs. Acetylation, deamidation, oxidation and phosphorylation were included as modifications in the protein quantification. Only phosphosites with a localisation probability of 0.75 or greater were retained for further analysis.

Data processing Time courses for the change in phosphorylation level for each phosphosite were calculated using the composite intensity scores estimated by MaxQuant from all peptide evidence for that site. For each time point (0, 6, 12, 18 and 60 s), these phosphosite intensities were quantile normalised between the four biological replicates Bolstad et al. (2003). For each site at each time point, the mean and standard error of the intensities were calculated if intensities were measured in at least two replicates, otherwise it was considered not to have been detected. The standard error of the site's intensities at a given time point was compared to the distribution of standard errors of all sites' intensities for that time point. If a site's standard error of intensities at any time point was greater than the distribution's upper quartile plus 1.5 times the interquartile range (IQR) (*i.e.* the upper "whisker" on a standard box-and-whisker plot), that site was discarded as unreliable.

Three time courses were then generated from these values: the "full" time course (0–60 s), for sites that were detected at all five time points; a "late" truncated time course (6–60 s), for sites that were not detected in unactivated parasites; and an "early" truncated time course (0–18 s), for sites that were not detected at the 60 s time point. For each site, ratios were calculated from its intensity at each time point against its intensity at the first time point and were logarithmically transformed (base 2) (*e.g.* $log_2\left(\frac{6\,s}{0\,s}\right)$). Only complete time courses, with no missing data, were retained for further analysis.

In order to determine which sites show significant evidence of change in phosphorylation state, we exploited the fact that enrichment of phosphopeptides is not 100 % specific and that the time-frame in question is too short to see significant variation in protein abundances. Time courses were constructed for non-phosphorylated peptides in the same manner as described above. All phosphosite ratios and non-phosphopeptide ratios were then corrected for the median non-phosphopeptide ratio for the corresponding time point. The distributions of non-phosphopeptide log_2 ratios were used as null distributions against which the significance of the phosphosite log_2 ratios could be tested. Two-tailed p-values were computed from an empirical cumulative distribution function of the non-phosphopeptide ratios. Sites showing at least one

time point with a p-value less than the critical value of 0.05 were determined to have undergone significant phosphoregulation during the time course.

Significant sites were clustered according to their time courses using a normal mixture-modeling based method, using the "mclust" library (version 5.2) in R (Fraley and Raftery, 2002; Fraley et al., 2012). The library chooses the number of clusters and the specific model to fit (spherical with equal volumes or spherical with unequal volumes) via an automated method which optimises the Bayesian Information Criterion (BIC).

Knock-out experiments

Phosphopeptide enrichment The CDPK4KO and the SRPK1KO experiments consisted of 8 samples each: wild-type and knock-out samples measured at two time points, unactivated (0 s) and activated (18 s after activation), in two biological replicates.

200 µL of 2 x lysis buffer was added to the 200 µL parasite suspension, and then processed as described above. 300 µg proteins were taken and the volumes were equalised with 100 mM TEAB followed by alkylation with IAA. Proteins were precipitated by MTBE (Matyash et al., 2008) and then digested with 8 µg trypsin in 150 µL of 100 mM TEAB at 37 °C for 2 h. A further 4 µg trypsin was added and digested for another 5 h. 100 µg peptides was taken from each replicate for TMT 10plex labelling, then mixed and dried in a SpeedVac. The labelled peptide mixture was fractionated at pH 10 on an XBridge BEH C18 column (4.6 mm id x 250 mm, 130 Å, 3.5 µm) (Waters) at a flow rate of 500 µL/min with a linear gradient from 5–35 % ACN/NH_3 in 30 min and total cycle time of 60 min. Fractions were collected every 30 s and between 2.5–50 min on a 96-well plate by rows and then concatenated into 12 fractions by columns and dried in a SpeedVac.

Enrichment of phosphopeptides was performed using IMAC with PHOS-Select Iron Affinity Gel (Sigma) then TiO_2 tips (Thermo Fisher) sequentially. All procedures followed the manufacturer's instruction with some modification. The peptides were redissolved in 50 % ACN/0.1 % TFA then added to 100 µL of pre-washed PHOS-Select Iron Affinity Gel and left binding at room temperature with end-to-end rotation for 30 min. The beads were washed three times with 250 mM acetic acid/50 % ACN and once with H_2O. Phosphopeptides were eluted twice with 100 µL of 1.5 % NH_3/25 % ACN then dried in a SpeedVac. The flow-through and the first wash of IMAC beads were collected and dried in a SpeedVac, and then the phosphopeptides were enriched using TiO_2 tips as described above.

TMT-labelled LC-MS/MS analysis The enriched phosphopeptides were redissolved in 0.5 % FA before LC-MS/MS analysis on an Orbitrap Fusion Tribrid mass spectrometer coupled with an Ultimate 3000 RSLCnano system configured as above. The peptides were separated with a linear gradient of 4–36 % ACN/0.1 % FA in 120 min and total 153 min per cycle. The Orbitrap Fusion was operated using the "Top Speed" method with 2 s cycle time. The MS full scan was in the Orbitrap with the following settings: scan range at m/z 380–1500 with a lock mass at 445.120 025, resolution at 120 000 at m/z 200, and AGC at 4×10^5 with a maximum injection time at 50 ms. The multiply-charged precursor ions (2+ to 6+) at most intense, with a minimal signal above 10 000 counts, were dynamically selected for high energy collision-induced dissociation (HCD) (MS/MS) and detected in the Orbitrap with a resolution at 30 000 at m/z 200. The isolation width was 1.2 Da in quadrupole, and the collision energy was set at 40 %. The dynamic exclusion duration time was set for 60 s with ±10 ppm exclusion mass width, the AGC was set at 1×10^5 with the maximum injection time at 105 ms.

The phosphopeptides enriched via IMAC and TiO_2 were analysed separately.

Spectral analysis and peptide quantification Raw data were processed in Proteome Discoverer 2.1 (PD2.1; Thermo Fisher) using both SequestHT and Mascot search engines against a combined protein database of *P. berghei* and mouse as above. Trypsin maximum missed cleavage sites were set to 2. The dynamic modifications set in both Mascot and SequestHT were Acetyl (N-term), Deamidated (NQ), Phospho

(STY) and Oxidation (M), while in SequestHT Camabidomethyl (C) was set as a fixed modification. The remaining settings were the same in both: precursor mass tolerance at 20 ppm, fragment at 0.5 Da, and TMT6plex as fixed modification. All files acquired from same set of TMT10plex experiments, including from both IMAC and TiO_2 purified samples, were set as fractions. The search result was validated by Percolate where the q-value was set at 0.01. The PSMs were identified, quantified and grouped to the peptides. Both PSMs and peptides were filtered with 1 % FDR, then further group to proteins with 1 % FDR where only peptides at high confidence were selected. Both unique and razor peptides were used for protein quantification, and protein and peptide abundance values were calculated as summed PSM quan values (S/N values of reporter ions). The abundances were normalised on Total Peptide Amount, and then scaled with On Channels Average. The co-isolation threshold was set at 50 % to reduce the isolation interference. The phosphorylation sites were localised by phosphoRS as implemented in PD2.1 with site probability set at 75.

Data processing For each knock-out experiment, peptides were filtered to include those which were unambiguously matched to a single *P. berghei* or *M. musculus* protein with a FDR less than 0.01. Furthermore, only peptides that were quantified in all TMT channels were retained. log_2 ratios were calculated for both biological replicates using the peptides' scaled abundances provided by Proteome Discoverer for the following comparisons: KO 18 s vs. KO 0 s, WT 18 s vs. WT 0 s, KO 18 s vs. WT 18 s, and KO 0 s vs. WT 0 s. For each comparison in each biological replicate, the ratios were corrected by subtracting the median value. Finally, for each comparison, a mean ratio was calculated by first computing the mean ratio within each replicate for each of the peptide's PSMs (*i.e.* those PSMs with phosphorylation at the same site(s) but otherwise differing in other modifications), followed by computing the mean of the resulting value between the two biological replicates. For all ratios, a p-value was calculated using the empirical cumulative distribution function of the non-phosphopeptides, as described above.

In order to account for a general change in cellular state before gametocyte activation, we built a linear model to predict the disruption at 18 s from the disruption at 0 s. That is, we aimed to predict $log_2\left(\frac{\text{KO 18 s}}{\text{WT 18 s}}\right)$ from $log_2\left(\frac{\text{KO 0 s}}{\text{WT 0 s}}\right)$. We reasoned that peptides which were poorly predicted by this model could be assumed to have been affected by the knock-out during the activation process. To quantify this, we Studentised the residuals and assigned p-values from the t-distribution. Peptides that were found to have both residual p-values and $log_2\left(\frac{\text{KO 18 s}}{\text{WT 18 s}}\right)$ ratio p-values less than 0.05 were taken to be significantly affected by the knock-out. We also performed a further partitioning of the affected peptides to isolate those which suffered an apparent loss of phospho-regulation, by taking those which were: a) observed to be significantly up-regulated in the WT sample or in an up-regulation cluster in the time-course experiment; and b) either significantly down-regulated or not significantly regulated in the KO sample.

Determination of gametocyte ploidy by FACS analysis

Ploidy of gametocytes was determined by FACS measurement of fluorescence intensity of cells stained with Vybrant dye cycle violet (life Technologies). Parasites were treated for two days with sulfadizine and gametocytes were purified and resuspended in 100 µL of SA. Activation was induced by adding 100 µL of modified exflagellation medium (RPMI 1640 containing 25 mM HEPES, 4 mM sodium bicarbonate, 5 % FCS, 200 µM xanthurenic acid, pH 7.8). To rapidly block gametogenesis, 800 µL of ice-cold PBS was added and cells were stained for 30 min at 4 °C with Vybrant dye cycle violet. Cells were analysed with a Beckman Coulter Gallios 4. Per sample, fluorescence intensity of >50 000 cells was determined with the Kaluza analysis software.

Immunofluorescence labelling and microscopy

Gametocyte immunofluorescence assays were performed as previously described (Volkmann et al., 2012). Purified cells were fixed with 4 % paraformaldehyde and 0.05 % glutaraldehyde in PBS for one hour, permeab-

ilised with 0.1 % Triton X-100/PBS for 10 min and blocked with 2 % BSA/PBS for 2 h. Mouse anti-α-tubulin clone DM1A (Sigma-Aldrich) primary antibodies were diluted 1:1000 in blocking solution. Anti-mouse Alexa488 was used as secondary antibody together with DAPI (all from Life technologies), all diluted 1:1000 in blocking solution. Confocal images were acquired with a LSM700 scanning confocal microscope (Zeiss).

Orthology

Protein orthology relationships were predicted using OMA (standalone version 0.99z.2) (Roth et al., 2008). The orthology predictions were performed on the proteomes of seven *Plasmodium* species: *P. berghei* ANKA, *P. chabaudi chabaudi*, *P. falciparum* 3D7, *P. knowlesi* H, *P. reichenowi* CDC, *P. vivax* Sal1, and *P. yoelii yoelii* 17XNL. Annotated protein sequences were fetched from PlasmoDB (version 13.0; `http://www.plasmodb.org`). OMA was run with a minimum alignment score of 181, an alignment length tolerance of 0.41, a stable-pair distance tolerance of 1.81, a verified-pair distance tolerance of 1.53 and a minimum sequence length of 50 residues.

Protein function and sex-specificity enrichment

Gene Ontology (GO) association files for *P. berghei* and *P. falciparum* (version 1.3.2015) were fetched from the Wellcome Trust Sanger Institute FTP server (`ftp://ftp.sanger.ac.uk`). The *P. falciparum* GO associations were used to supplement the *P. berghei* ones. For each *P. falciparum* gene with a one-to-one ortholog in *P. berghei*, the corresponding *P. falciparum* terms were merged with the existing terms associated with the *P. berghei* ortholog (if any).

Groups of proteins were tested for enrichment of GO terms via a binomial test (Mi et al., 2013). Query protein groups for the time-course experiment included the list of all proteins containing significant sites and the lists of proteins containing significant sites in each of the phosphosite clusters. For the knock-out experiments, the query groups included proteins that showed significant up- or down-regulation in the wild-type sample, those whose post-activation state was significantly disrupted in the knock-out versus the wild-type, and those which we determined to be putative down-stream targets of the deleted kinase. The "background" set of proteins, against which enrichment was tested, consisted of all of the proteins detected in the time-course mass-spectrometry experiments, including those detected via non-phosphorylated peptides. GO enrichment analyses were performed using GOATOOLS (version 0.5.9) (Tang et al., 2015), modified to perform the binomial test, on the "go-basic" ontology file provided at `http://purl.obolibrary.org/obo/go/go-basic.obo`. For the knock-out experiments, we only performed targeted enrichment analyses by testing only the "leaf" terms found enriched in the time-course experiment.

Groups of proteins were further tested for enrichment for sex-specific proteins, using a previously published *P. berghei* sex-partitioning data set (Tao et al., 2014), which was itself a reanalysis of sex-specific proteomics data (Khan et al., 2005). Fisher's exact test was used to determine significant enrichment for female-specific, male-specific or shared proteins.

Network analysis

Protein association networks for *P. berghei* and *P. falciparum* were downloaded from the STRING database (version 10; `http://www.string-db.org`) (Szklarczyk et al., 2015). The *P. berghei* network was extended to include additional nodes and edges from the *P. falciparum* network by orthology. New *P. berghei* nodes were added if they have a one-to-one ortholog in the *P. falciparum* proteome; edges from the *P. falciparum* STRING network were added to the *P. berghei* network only in the case that the edge was not already present. Network statistics were computed using the NetworkX library for Python (version 1.8.1) (Hagberg et al., 2008).

Significant associations on the network between protein kinases or phosphatases and the phosphosite time-course clusters were determined through enrichment analyses for each phosphoregulated enzyme. We also tested enzymes that have previously been implicated in gametogenesis (Figure 4). We compared the median of all of a kinase's edge scores with the median of its scores with the proteins of a given time course cluster. We tested the hypothesis that the median edge score for the subset of proteins with sites in the cluster was higher than that of all the enzyme's edges via the Mann-Whitney test.

Protein Domain Prediction

The presence of Pfam domains in *P. berghei* proteins was predicted using the "pfamscan" script (upload date 2013-10-15) available on the Wellcome Trust Sanger Institute FTP server (`ftp://ftp.sanger.ac.uk/pub/databases/Pfam/Tools`). The script performed sequence searches using version 3.1b1 of HMMR Eddy (2011) and version 27.0 of the Pfam database (Finn et al., 2016).

Site Disorder

Site disorder estimates were calculated using DISOPRED (version 3.16) (Jones and Ward, 2003). DISOPRED performed PSI-BLAST searches using the BLAST executable "blastpgp" (version 2.2.26) on the "nr" database with the default parameters specified in the script provided by the DISOPRED software package.

Site Conservation

Site conservation was measured by fitting nucleotide alignments of orthologous genes with site-specific codon substitution models. An estimate of the ratio of the number of non-synonymous substitutions per site to the number of synonymous substitutions per site (dN/dS) during species divergence was used as a measurement of conservation; dN/dS values less than one indicate that the site has predominantly been under purifying selection, with lower values indicating stronger selective constraint and conservation.

A nucleotide multiple-sequence alignment was produced for the genes in each orthologous group using PRANK (version .140110), aligning by codons (option "-codon") and leaving sites inferred as insertions unaligned (option "-F") (Löytynoja and Goldman, 2005; 2008). Regions with gaps were automatically removed using TrimAL (Capella-Gutiérrez et al., 2009). In order to estimate a species tree for the conservation analysis, a composite alignment was built by concatenating the 3150 alignments that contained orthologs from all seven species. The tree was estimated using PhyML with the GTR nucleotide substitution model and the better of the NNI and SPR tree topology search methods (option "-s BEST") (Guindon et al., 2010).

Site dN/dS estimates were computed using the site models implemented in the "codeml" program of the PAML package (version 4.8a) (Yang, 2007). The analyses were automated using the PAML interface implemented in the Bio.Phylo module of Biopython (Talevich et al., 2012). In order to distinguish between positive and purifying selection, each alignment was fitted with a pair of models, termed M8 and M8a (Swanson et al., 2003; Wong et al., 2004). These models are nested, permitting the better model to be chosen via a likelihood-ratio test. Model M8a assigns sites to classes either with an estimated $0 < dN/dS < 1$ drawn from the β-distribution (purifying selection) or with $dN/dS = 1$ (neutral evolution). Model M8 is similar, albeit with the latter category estimated with $dN/dS > 1$ (positive selection). The equilibrium codon frequencies in the models were estimated from the average nucleotide frequencies at the three codon positions. The parameter κ was estimated from an initial value of 4.0, the parameter α was fixed at infinity, and the parameter ρ was fixed at 0. The likelihood-ratio tests were performed with one degree of freedom using twice the log-likelihood difference of the two models on the χ^2_1 distribution (Wong et al., 2004); a significant test would indicate that the M8 model, featuring positively selected sites, better fits the alignment than the null model, M8a. p-values were adjusted for FDR and a critical value of 0.05 was used to determine significance. dN/dS estimates for specific sites on a given protein (*e.g.* significant phosphosites) were taken from the

Naive Empirical Bayes (M8a) or Bayes Empirical Bayes (M8) site-rate predictions for the chosen model, as produced by the software.

Subsequent analyses of site conservation included only sites under purifying selection ($dN/dS < 1$). Because we focus only on sites with $0 < dN/dS < 1$, the data is best represented by the beta distribution. A regression model of dN/dS scores was built using beta regression, as implemented in the "betareg" library for R (version 3.0-5) (Cribari-Neto and Zeileis, 2010). Two terms are modelled: the mean and a precision parameter, ϕ, which accounts for heteroskedasticity. After model reduction, the coefficients in the model of the mean were: DISOPRED score; median dN/dS score for the twenty sites preceding and twenty sites following the site, not including the three nearest positions on either side; the log_{10}-transformed median expression level of the RNA encoding the protein (Otto et al., 2014); whether or not the site is significantly regulated; and an interaction between the region median dN/dS and the median expression level terms. The mean model was fit using the logit link function. The model of the precision parameter included the following coefficients: DISOPRED score, log_{10}-transformed median RNA expression level, and whether or not the site is significantly regulated. All terms were significant at a level of $p < 0.001$.

Motor Protein Phosphorylation Hot-Spots

We identified and grouped proteins belonging to the kinesin, dynein heavy chain, dynein light intermediate chain and dynein intermediate chain families according to their annotations in the proteome databases for *P. berghei*, *P. falciparum, T. gondii*, *M. musculus*, *Homo sapiens*, *Rattus norvegicus* and *Saccharomyces cerevisiae*. In addition, we considered a group of *Plasmodium* proteins for which no known function has been described (denoted as "conserved *Plasmodium* protein, unknown function"), but which have been assigned the "motor activity" GO term (GO:0003774) in *P. falciparum* at PlasmoDB. We performed orthology predictions as described above for all of these protein families. Several of the unannotated *Plasmodium* proteins were found to be members of a hierarchical orthology group which contained annotated kinesin-like proteins, including the human proteins KIF15 and KIF20B: PBANKA_123650, PBANKA_093930, PBANKA_092720, PBANKA_121680, PBANKA_112530, and PBANKA_124420. We thus treated these proteins and their *P. falciparum* orthologs as belonging to the kinesin family. Previously published human, mouse and rat phosphosite information was retrieved from Phosphosite Plus (Hornbeck et al., 2015) (date of data set: 2016 Jan. 29). For yeast, phosphosite data was retrieved from Phosphogrid (Sadowski et al., 2013) (downloaded 2014 Mar. 14). Phosphosite data was manually curated for *T. gondii* (Treeck et al., 2011; 2014), *P. falciparum* (Solyakov et al., 2011; Treeck et al., 2011; Lasonder et al., 2012; Pease et al., 2013; Collins et al., 2014; Lasonder et al., 2015) and *P. berghei* (Brochet et al., 2014). The *P. berghei* was further supplemented with the phosphosites observed in our time course experiment.

A multiple-sequence alignment was computed for each family using MAFFT (version 7.205) (Katoh and Standley, 2013), using the "E-INS-i" methodology, which is "suitable for sequences containing large unalignable regions" (options "`--ep 0 --genafpair --maxiterate 1000`"). Alignment quality was calculated using Jalview (version 2.9.0b2) (Waterhouse et al., 2009), normalised by the maximum quality, and a running mean was calculated using a window of length 15. Hot-spots were estimated by performing a kernel density estimation on the phosphosite counts at each column in the alignment, using the Epanechnikov kernel and using the Sheather & Jones ("SJ") algorithm for automatic bandwidth selection (Sheather and Jones, 1991), as implemented in R.

Network Visualisation

Network visualisations were produced using Ti*k*Z and LaTeX based on a custom modification of the Walshaw 2000 spring-electrical layout algorithm (Walshaw, 2000; 2003; Tantau, 2013), using a spring constant of 0.1, a convergence tolerance of 0.001, an electric force order of 4, an electric charge of 0.085, and a cooling factor of 1.0. Phosphosite nodes were added to protein nodes as a sub-layout using the same algorithm with a

spring constant of 1.5 and an electric force order of 1 (fewer than nine phosphosites) or 0 (nine or more phosphosites).

References

Billker, O., Dechamps, S., Tewari, R., Wenig, G., Franke-Fayard, B., and Brinkmann, V. (2004). Calcium and a calcium-dependent protein kinase regulate gamete formation and mosquito transmission in a malaria parasite. Cell *117*, 503–514.

Bolstad, B.M., Irizarry, R.A., Astrand, M., and Speed, T.P. (2003). A comparison of normalization methods for high density oligonucleotide array data based on variance and bias. Bioinformatics *19*, 185–193.

Brochet, M., Collins, M.O., Smith, T.K., Thompson, E., Sebastian, S., Volkmann, K., Schwach, F., Chappell, L., Gomes, A.R., Berriman, M., et al. (2014). Phosphoinositide Metabolism Links cGMP-Dependent Protein Kinase G to Essential Ca^{2+} Signals at Key Decision Points in the Life Cycle of Malaria Parasites. PLoS Biol. *12*, e1001806.

Capella-Gutiérrez, S., Silla-Martínez, J.M., and Gabaldón, T. (2009). TrimAl: A tool for automated alignment trimming in large-scale phylogenetic analyses. Bioinformatics *25*, 1972–1973.

Collins, M.O., Wright, J.C., Jones, M., Rayner, J.C., and Choudhary, J.S. (2014). Confident and sensitive phosphoproteomics using combinations of collision induced dissociation and electron transfer dissociation. J. Proteomics *103*, 1–14.

Cox, J. and Mann, M. (2008). MaxQuant enables high peptide identification rates, individualized p.P.B.-Range mass accuracies and proteome-wide protein quantification. Nat. Biotechnol. *26*, 1367–1372.

Cribari-Neto, F. and Zeileis, A. (2010). Beta regression in R. Journal of Statistical Software *34*, 1–24.

Eddy, S.R. (2011). Accelerated Profile HMM Searches. PLoS Comput. Biol. *7*, e1002195.

Fang, H., Klages, N., Baechler, B., Hillner, E., Yu, L., Pardo, M., Choudhary, J., and Brochet, M. (2017). Multiple short windows of calcium-dependent protein kinase 4 activity coordinate distinct cell cycle events during *Plasmodium* gametogenesis. eLife *6*, e26524.

Finn, R.D., Coggill, P., Eberhardt, R.Y., Eddy, S.R., Mistry, J., Mitchell, A.L., Potter, S.C., Punta, M., Qureshi, M., Sangrador-Vegas, A., et al. (2016). The Pfam protein families database: Towards a more sustainable future. Nucleic Acids Research *44*, D279–85.

Fraley, C. and Raftery, A.E. (2002). Model-based clustering, discriminant analysis, and density estimation. Journal of the American Statistical Association *97*, 611–631.

Fraley, C., Raftery, A.E., and Scrucca, L. (2012). mclust version 4 for r: Normal mixture modeling for model-based clustering, classification, and density estimation. Technical Report No. 597, Department of Statistics, University of Washington. .

Guindon, S., Dufayard, J.F., Lefort, V., Anisimova, M., Hordijk, W., and Gascuel, O. (2010). New algorithms and methods to estimate maximum-likelihood phylogenies: Assessing the performance of PhyML 3.0. Syst. Biol. *59*, 307–321.

Hagberg, A.A., Schult, D.A., and Swart, P.J. (2008). Exploring network structure, dynamics, and function using NetworkX. In Proceedings of the 7th Python in Science Conference (Los Alamos National Laboratory (LANL)), pages 11–15. SciPy.

Hornbeck, P.V., Zhang, B., Murray, B., Kornhauser, J.M., Latham, V., and Skrzypek, E. (2015). PhosphoSitePlus, 2014: Mutations, PTMs and recalibrations. Nucleic Acids Research *43*, D512–20.

Jones, D.T. and Ward, J.J. (2003). Prediction of disordered regions in proteins from position specific score matrices. Proteins: Structure, Function, and Bioinformatics *53–Suppl–6*, 573–578.

Katoh, K. and Standley, D.M. (2013). MAFFT multiple sequence alignment software version 7: Improvements in performance and usability. Mol. Biol. Evol. *30*, 772–780.

Khan, S.M., Franke-Fayard, B., Mair, G.R., Lasonder, E., Janse, C.J., Mann, M., and Waters, A.P. (2005). Proteome analysis of separated male and female gametocytes reveals novel sex-specific *Plasmodium* biology. Cell *121*, 675–687.

Lasonder, E., Green, J.L., Camarda, G., Talabani, H., Holder, A.A., Langsley, G., and Alano, P. (2012). The *Plasmodium falciparum* schizont phosphoproteome reveals extensive phosphatidylinositol and cAMP-protein kinase A signaling. Journal of Proteome Research *11*, 5323–5337.

Lasonder, E., Green, J.L., Grainger, M., Langsley, G., and Holder, A.A. (2015). Extensive differential protein phosphorylation as intraerythrocytic *Plasmodium falciparum* schizonts develop into extracellular invasive merozoites. Proteomics *15*, 2716–2729.

Löytynoja, A. and Goldman, N. (2005). An algorithm for progressive multiple alignment of sequences with insertions. Proc. Natl. Acad. Sci. USA *102*, 10557–10562.

Löytynoja, A. and Goldman, N. (2008). Phylogeny-aware gap placement prevents errors in sequence alignment and evolutionary analysis. Science *320*, 1632–1635.

Matyash, V., Liebisch, G., Kurzchalia, T.V., Shevchenko, A., and Schwudke, D. (2008). Lipid extraction by methyl-tert-butyl ether for high-throughput lipidomics. Journal of Lipid Research *49*, 1137–1146.

Mi, H., Muruganujan, A., Casagrande, J.T., and Thomas, P.D. (2013). Large-scale gene function analysis with the PANTHER classification system. Nature Protocols *8*, 1551–1566.

Otto, T.D., Böhme, U., Jackson, A.P., Hunt, M., Franke-Fayard, B., Hoeijmakers, W.A.M., Religa, A.A., Robertson, L., Sanders, M., Ogun, S.A., et al. (2014). A comprehensive evaluation of rodent malaria parasite genomes and gene expression. BMC Biol. *12*, 86.

Pease, B.N., Huttlin, E.L., Jedrychowski, M.P., Talevich, E., Harmon, J., Dillman, T., Kannan, N., Doerig, C., Chakrabarti, R., Gygi, S.P., et al. (2013). Global analysis of protein expression and phosphorylation of three stages of *Plasmodium falciparum* intraerythrocytic development. Journal of Proteome Research *12*, 4028–4045.

Roth, A.C.J., Gonnet, G.H., and Dessimoz, C. (2008). Algorithm of OMA for large-scale orthology inference. BioMed Central Bioinformatics *9*, 518.

Sadowski, I., Breitkreutz, B.J., Stark, C., Su, T.C., Dahabieh, M., Raithatha, S., Bernhard, W., Oughtred, R., Dolinski, K., Barreto, K., et al. (2013). The PhosphoGRID Saccharomyces cerevisiae protein phosphorylation site database: Version 2.0 Update. Database (Oxford) *2013*, bat026.

Schwanhäusser, B., Busse, D., Li, N., Dittmar, G., Schuchhardt, J., Wolf, J., Chen, W., and Selbach, M. (2011). Global quantification of mammalian gene expression control. Nature *473*, 337–342.

Sheather, S.J. and Jones, M.C. (1991). A reliable data-based bandwidth selection method for kernel density estimation. Journal of the Royal Statistical Society. Series B (Methodological) pages 683–690.

Solyakov, L., Halbert, J., Alam, M.M., Semblat, J.P., Dorin-Semblat, D., Reininger, L., Bottrill, A.R., Mistry, S., Abdi, A., Fennell, C., et al. (2011). Global kinomic and phospho-proteomic analyses of the human malaria parasite *Plasmodium falciparum*. Nat Commun *2*, 565.

Swanson, W.J., Nielsen, R., and Yang, Q. (2003). Pervasive adaptive evolution in mammalian fertilization proteins. Mol. Biol. Evol. *20*, 18–20.

Szklarczyk, D., Franceschini, A., Wyder, S., Forslund, K., Heller, D., Huerta-Cepas, J., Simonovic, M., Roth, A., Santos, A., Tsafou, K.P., et al. (2015). STRING v10: Protein-protein interaction networks, integrated over the tree of life. Nucleic Acids Research *43*, D447–52.

Talevich, E., Invergo, B.M., Cock, P.J., and Chapman, B.A. (2012). Bio.Phylo: A unified toolkit for processing, analyzing and visualizing phylogenetic trees in Biopython. BMC Bioinformatics *13*, 209.

Tang, H., Klopfenstein, D., Pedersen, B., Flick, P., Sato, K., Ramirez, F., Yunes, J., and Mungall, C. (2015). Goatools: Tools for gene ontology DOI: 10.5281/zenodo.31628.

Tantau, T. (2013). Graph Drawing in TikZ. Journal of Graph Algorithms and Applications *17*, 495–513.

Tao, D., Ubaida-Mohien, C., Mathias, D.K., King, J.G., Pastrana-Mena, R., Tripathi, A., Goldowitz, I., Graham, D.R., Moss, E., Marti, M., et al. (2014). Sex-partitioning of the *Plasmodium falciparum* stage V gametocyte proteome provides insight into falciparum-specific cell biology. Mol. Cell Proteomics *13*, 2705–2724.

Tewari, R., Straschil, U., Bateman, A., Böhme, U., Cherevach, I., Gong, P., Pain, A., and Billker, O. (2010). The systematic functional analysis of *Plasmodium* protein kinases identifies essential regulators of mosquito transmission. Cell Host Microbe *8*, 377–387.

Treeck, M., Sanders, J.L., Elias, J.E., and Boothroyd, J.C. (2011). The phosphoproteomes of *Plasmodium falciparum* and *Toxoplasma gondii* reveal unusual adaptations within and beyond the parasites' boundaries. Cell Host Microbe *10*, 410–419.

Treeck, M., Sanders, J.L., Gaji, R.Y., LaFavers, K.A., Child, M.A., Arrizabalaga, G., Elias, J.E., and Boothroyd, J.C. (2014). The calcium-dependent protein kinase 3 of toxoplasma influences basal calcium levels and functions beyond egress as revealed by quantitative phosphoproteome analysis. PLoS Pathog. *10*, e1004197.

Vincke, I.H., Bafort, J., and Scheepers-Biva, M. (1966). Recent observations on the cyclic transmission of *Plasmodium berghei*. Ann. Soc. Belges. Med. Trop. Parasitol. Mycol. *46*, 327–336.

Volkmann, K., Pfander, C., Burstroem, C., Ahras, M., Goulding, D., Rayner, J.C., Frischknecht, F., Billker, O., and Brochet, M. (2012). The alveolin IMC1h is required for normal ookinete and sporozoite motility behaviour and host colonisation in *Plasmodium berghei.* PLoS ONE *7*, e41409.

Walshaw, C. (2000). A multilevel algorithm for force-directed graph drawing. In Graph Drawing (Springer), pages 171–182.

Walshaw, C. (2003). A multilevel algorithm for force-directed graph-drawing. Journal of Graph Algorithms and Applications *7*, 253–255.

Waterhouse, A.M., Procter, J.B., Martin, D.M.A., Clamp, M., and Barton, G.J. (2009). Jalview Version 2–a multiple sequence alignment editor and analysis workbench. Bioinformatics *25*, 1189–1191.

Wiśniewski, J.R., Zougman, A., Nagaraj, N., and Mann, M. (2009). Universal sample preparation method for proteome analysis. Nat. Methods *6*, 359–362.

Wong, W.S.W., Yang, Z., Goldman, N., and Nielsen, R. (2004). Accuracy and power of statistical methods for detecting adaptive evolution in protein coding sequences and for identifying positively selected sites. Genetics *168*, 1041–1051.

Yang, Z. (2007). PAML 4: phylogenetic analysis by maximum likelihood. Mol. Biol. Evol. *24*, 1586–1591.
